# Dietary regulation in health and disease

**DOI:** 10.1038/s41392-022-01104-w

**Published:** 2022-07-23

**Authors:** Qi Wu, Zhi-Jie Gao, Xin Yu, Ping Wang

**Affiliations:** 1 Tongji University Cancer Center, Shanghai Tenth People’s Hospital of Tongji University, School of Medicine, Tongji University, Shanghai, 200092 China; 2 Department of Breast and Thyroid Surgery, Renmin Hospital of Wuhan University, Wuhan, Hubei P. R. China

**Keywords:** Predictive markers, Translational research

## Abstract

Nutriments have been deemed to impact all physiopathologic processes. Recent evidences in molecular medicine and clinical trials have demonstrated that adequate nutrition treatments are the golden criterion for extending healthspan and delaying ageing in various species such as yeast, drosophila, rodent, primate and human. It emerges to develop the precision-nutrition therapeutics to slow age-related biological processes and treat diverse diseases. However, the nutritive advantages frequently diversify among individuals as well as organs and tissues, which brings challenges in this field. In this review, we summarize the different forms of dietary interventions extensively prescribed for healthspan improvement and disease treatment in pre-clinical or clinical. We discuss the nutrient-mediated mechanisms including metabolic regulators, nutritive metabolism pathways, epigenetic mechanisms and circadian clocks. Comparably, we describe diet-responsive effectors by which dietary interventions influence the endocrinic, immunological, microbial and neural states responsible for improving health and preventing multiple diseases in humans. Furthermore, we expatiate diverse patterns of dietotheroapies, including different fasting, calorie-restricted diet, ketogenic diet, high-fibre diet, plants-based diet, protein restriction diet or diet with specific reduction in amino acids or microelements, potentially affecting the health and morbid states. Altogether, we emphasize the profound nutritional therapy, and highlight the crosstalk among explored mechanisms and critical factors to develop individualized therapeutic approaches and predictors.

## Introduction

What we eat and how this may influence our health has aroused interest for millennia. In ancient times, wild animals and foraged plants exclusively were acquired as foods, and diet fluctuated with the seasons.^1^ Currently, we can obtain food continually due to advanced technology in agriculture and animal husbandry. As a consequence, the impact of nutrition on humans has intrinsically diverged in human evolution. In many cases, diets are inundated with excessive amounts of calories, highly processed foods and a mass of salt, trans fat and sugar.^2^ Hence, dramatic changes in the quantity, quality, and frequency of foods are considered to result in human maladaptation.

**Fig. 1:**
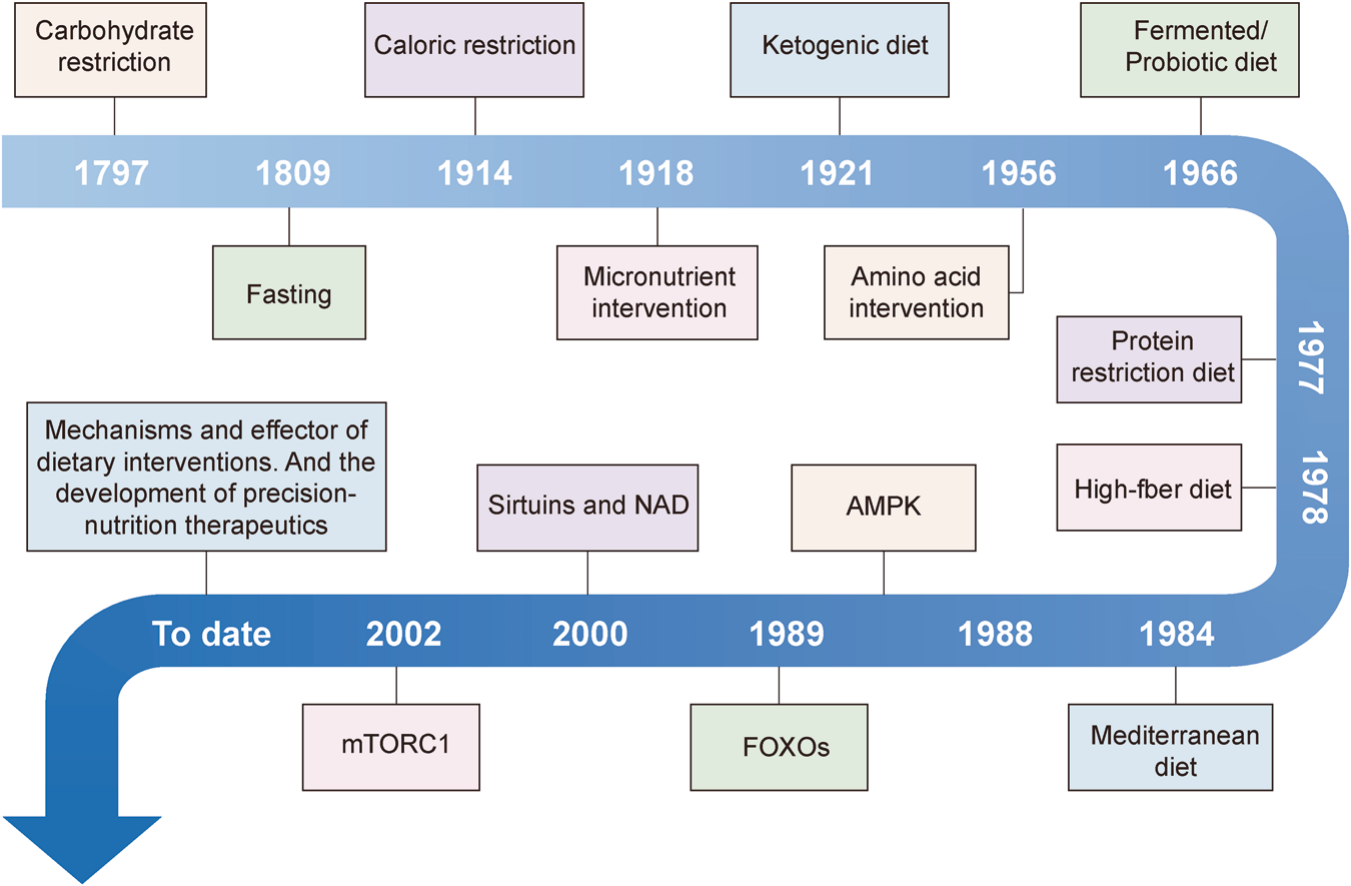
Research history of dietary regulation. The advances in dietary regulation can be roughly divided into three stages. Stage I, dietary regulation discovery and initial characterizations of their effects on body function, including dietary interventions based on traditional eating habits, such as fasting,^367^ caloric restriction,^368^ high-fibre diet,^369^ fermented/probiotic diet,^370^ Mediterranean diet^371^ and protein restriction,^372^ some new dietary interventions, such as ketogenic diet,^373^ glucose and carbohydrate restriction,^374^ amino acid intervention^375^ and micronutrient intervention.^376,377^ Stage II, key molecular mechanisms of dietary interventions were identified, including mTORC1,^378^ AMPK,^379^ FOXOs^380^ and Sirtuins and NAD.^381^ To date, whether and how metabolic interventions interfere with these signalling pathways to function has been extensively studied. Stage III, dietary interventions have been being explored for their impact on the overall metabolic network of the body to develop precision-nutrition therapeutics

Nutrition influences all physiological processes. Calorie restriction (CR) without malnutrition was put forward long time ago, and was demonstrated to prolong lifespan in rats in 1935 (Fig. 1). However, the mechanisms by which CR improves healthspan and longevity have remained elusive until a few decades ago. First, mutations in a single gene involved in nutrient-sensing signalling pathways are found to substantially lengthen the lifespans of nematode worms.^3^ Consequently, nutrition-mediated mechanisms increasing healthspan and lifespan are discovered.^4^ In the clinical interpretation of these findings, conscious variation of nutrition has been advocated as a promising method for enhancing healthspan and safeguarding against a multitude of diseases such as cancer, obesity and Alzheimer disease (AD). The concept that diet could affect the risks of developing certain diseases and impact therapeutic effects has prevailed.^5^ It is unfortunate that the effectiveness of most of these diets has not been rigorously evaluated, and viewpoints in this realm are not always based on solid mechanistic insight.

The quantity, quality and composition of foods as well as meal timing directly impact our healthspan by regulating nutrient availability (Table 1). Despite varying dietary strategies, many sets of dietary guidelines have already been reported to be capable of extending lifetime in at least one model organism and improving health conditions in humans. Considering the dissimilarity across populations and individuals, variation in responses to diverse regimens is practical to anticipate. Precision nutrition, which means establishing personalized dietary plans to promote health, has lately been suggested as a novel strategy.^6^ Thus, many companies have attempted to implement precision nutrition to maximize lifespan while failing to maximize individual benefits by incorporating omics-based signatures.

**Table 1:** Types of dietary interventions

Dietary intervention		Description
Fasting	Classical	Starvation for random 2 days.
	Prolonged	Starvation for random >2 days.
	Intermittent	The alternate pattern of ad libitum food intake-encompassing regimes that may include alternate-day fasting (ADF), modified ADF (limited calories supplied during fasting day), or 5:2 diet (days of caloric restriction per week).
	Time-restricted	The time-limited fasting during a period of several hours per day.
Caloric restriction	Classical	Reduced caloric intake (20–30% below average) without undergoing malnutrition during the entire period of dietary intervention.
	Fasting-mimicking	Four days of diet that mimics fasting (FMD) consisting of very low calorie/low protein. The ad libitum diet is fed between the period of FMD cycles.
	Time-restricted	The time-limited consumption of calories during a period of several hours per day
Ketogenic diet		High-fat, moderate-protein and low-carbohydrate (usually <40 g/day) diets. The fat ingredients including saturated fatty acid (SFA), monounsaturated fatty acid (MUFA) and polyunsaturated fatty acids (PUFA) vary in different studies.
Glucose and carbohydrate restriction		Carbohydrate consumption is restricted relative to the average diet and is replaced by food containing a higher percentage of fat and protein. Glucose restriction refers to specific restriction of glucose intake instead of other forms of complex carbohydrates and sweeteners.
High-fibre diet/short chain fatty acids (SCFAs) supplements		Soluble dietary fibre (20 g/day) mainly by intake of fruits, vegetables, legumes and whole grain to produce SCFAs.
Fermented/probiotic diet		Commercial prebiotics, yogurt, matured cheese.
Plants-based diets/Mediterranean diet		Food mainly consists of fruits, vegetables, legumes, beans, olive oil and nuts but reduce meat consumption.
Protein restriction		Reduction of dietary protein intake without changing the average caloric intake.
Amino acid intervention		Specific restriction or supplement of amino acids including serine, glycine, tryptophan, histidine, lysine, methionine, threonine and branched-chain amino acid (leucine, isoleucine and valine).
Micronutrient intervention		Applicable intervention of vitamins and minerals supplement such as low-salt diet.
Metabolite intervention		Reduction or inhibiting biosynthesis of specific reaction intermediates or end-products of physiological metabolism, including N-acylethanolamines, folate metabolism intermediates, tricarboxylic cycle intermediates and coenzyme Q.

In this review, we detail the nutritional molecular mechanisms that result in improved health. We also propose to decipher factors associated with sex, genetics and age that affect responses to dietary intervention. Altogether, we develop an approach to precision-nutrition medicine to elucidate the health-promoting effects of diet-based therapeutics.

## Nutrient-associated molecular mechanisms

**Fig. 2:**
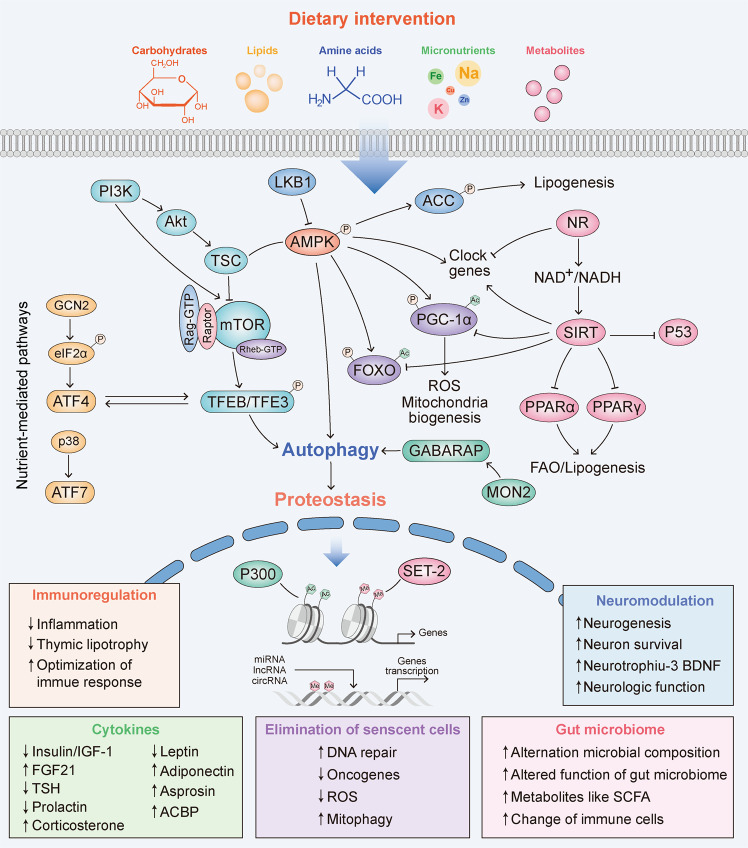
Molecular and effector mechanisms of dietary intervention. Dietary intervention engages by alterable consumption of numerous nutrients, including carbohydrates, lipids, amino acids, micronutrients, and metabolites. Nutrient signals under diverse dietary interventions lead to activation of multiple biochemical pathways. As a result, these pathways involve in downstream effectors like cytokines secretion, immunoregulation, gut microbiome homoeostasis, elimination of senescent cells, and neuromodulation

The mechanisms that are universally attributed to the effects of diet on disease and health are mainly classified into two types (Table 2; Fig. 2): (1) nutrient-mediated mechanisms, covering metabolic regulators, nutritive metabolism pathways, epigenetic mechanisms and circadian clocks; and (2) diet-responsive effectors, including the diet-endocrine axis, the diet-immune axis, the diet-gut axis, the diet-senescence axis and the diet-nerve axis.

**Table 2:** The mechanisms and effectors of dietary interventions

Dietary intervention	Pathway	Species	Effect	Reference
Fasting	–	Human	↓ fat mass, ↓ cholesterol, ↑ fatty acids, ↑ β-hydroxybutyrate	Stekovic et al.^80^
	TORC1	Fly	↑ autophagy, ↓ cysteine, ↑ acetyl-coenzyme A metabolism	Jouandin et al.^92^
	Oxidative stress resistance	Yeast	↑ lifespan	Brandhorst et al.^238^
	RHEB-1, IGF, DAF-16	Worm	↑ lifespan	Honjoh et al.^382^
	TOR independent	Fly	↑ improved gut health	Catterson et al.^383^
	IGF-1, PKA	Mouse	↑ lifespan, ↑ rejuvenated immune system, ↓ visceral fat, ↓ cancer incidence, ↓ skin lesions, ↓ bone mineral density loss	Brandhorst et al.^238^
	SIRT5	Mouse	↑ NAD^+^ level in liver, ↑ amino acid catabolism	Nakagawa et al.^42^
	–	Human	↑ fatty acids and β-hydroxybutyrate	Stekovic et al.^80^
	Circadian regulation and autophagy	Fly	↑ lifespan	Ulgherait et al.^87^
	MON-2	Worm	↑ autophagy, ↑ lifespan	Jung et al.^89^
	–	Human	↑ insulin sensitivity, ↓ body mass and adiposity, ↓ inflammation, ↑ gut microbial diversity	Xie et al.^147^
	BMAL1-PPARα	Mouse	↓ body temperature, ↑ hepatic NADH level	Levine et al.^148^
	p38-ATF7	Worm	↓ insulin/IGF-1, ↑ innate immunity, ↑ lifespan	Wu et al.^178^
	–	Human	alteration of gut microbiome and immune cells, ↓ systolic blood pressure	Maifeld et al.^196^
	–	Mouse	↑ neurotrophiω-3, ↑ brain-derived neurotrophic factor	Lee et al.^223^
Caloric restriction	mTOR	Yeast	↑ lifespan	Kaeberlein et al.^384^
		Worm	↑ autophagy	Hansen et al.^385^ Kapahi et al.^21^
		Fly	↓ protein synthesis, ↑ stress resistance, ↑ lifespan	Kapahi et al.^21^
	DAF-16/FOXO	Worm	↑ lifespan	Greer et al.^27^
	dFOXO independent	Fly	↑ lifespan	Giannakou et al.^28^
	FOXO1	Mouse	↓ inflammation, ↓ liver injury	Miyauchi et al.^29^
	FOXO3, FOXO4	Rat	↓ age-associated muscles dysfunction	Furuyama et al.^30^
	FOXO3	Mouse	↑ lifespan, ↓ cancer incidence	Shimokawa et al.^31^
	SIRT1	Worm	↑ lifespan	Morselli et al.^123^
		Rat	↑ lifespan	Cohen et al.^38^
		Young non-obese human	↑ muscle mitochondrial function	Civitarese et al.^40^
	SIRT3	Mouse	↑ NADPH, ↑ glutathione	Someya et al.^56^
		Mouse	↑ SOD2, ↓ ROS and oxidative stress	Qiu et al.^101^
		Mouse	↓ mitochondrial protein acetylation	Hebert et al.^57^
		Mouse	↑ mitochondrial glutathione antioxidant defense system	Someya et al.^56^
	SIR2, NPT1	Yeast	↑ lifespan	Lin et al.^36^
		Worm	↑ oxidative metabolism, ↑ lifespan	Moroz et al.^61^
	ER stress	Worm	↑ ER-UPR, ↑ proteostasis, ↑ lifespan	Matai et al.^84^
	AMPK	Worm	↑ FAO, ↑ peroxisomal function ↑ mitochondrial network homoeostasis, ↑ lifespan	Weir et al.^24^
	AKH	Fly	↑ fatty-acid synthesis and breakdown, ↑ lifespan	Katewa et al.^75^
	–	Mouse	↓ FA intake, ↑ fatty-acid synthesis, ↑ FAO	Bruss et al.^76^
	P38	Worm	↑ PUFAs, especially LA and EPA	Chamoli et al.^82^
	PGC-1α	Mouse	↑ mitochondrial biogenesis and function, ↓ ROS	López-Lluch et al.^102^
	–	Mouse	↓ oxidant emission, ↑ antioxidant scavenging, ↓ oxidative damage	Lanza et al.^103^
	miR-144/Nrf2	Rat	↓ inflammation, ↑ cerebrovascular function	Csiszar et al.^133^
	Tim	Fly	↑ lifespan	Katewa et al.^146^
	–	Mouse Human	↓ inflammation, ↑ reversed the aging-disturbed immune ecosystem	Ma et al.^179^
	PLA2G7	Mouse Human	↓ thymic lipoatrophy, ↓ inflammation, ↑ metabolic health	Spadaro et al.^100^
	Bacterial lipid A synthetase	Mouse	↓ inflammation and proinflammatory immune cells, ↓ fatty liver, ↑ beige fat	Fabbiano et al.^193^
	Clostridioides difficile	Mouse Human	↓ body weight, ↑ metabolic improvement	von Schwartzenberg et al.^192^
	–	Human	↑ alpha diversity of the gut microbiota, ↓ intestinal effector memory CD8^+^ T cells, ↓ intestinal memory B cells, ↓ hepatic effector memory CD4^+^, ↓ CD8^+^ T cells	Sbierski-Kind et al.^197^
	–	Mouse	↑ neurogenesis	Bondolfi et al.^221^ Weng et al.^386^ Lee et al.^223^
*Lipid-associated diet*				
Ketogenic diet	–	Mouse	↓ Th17 cells, ↓ bifidobacterial growth	Ang et al.^200^
	Hcar2	Mouse Human	↓ ISCs function, ↓ tumorigenesis	Dmitrieva-Posocco et al.^353^
	H3K9, PGC-1α, FOXO1	Mouse	↑ pentose phosphate and glycogen, ↑ T-cell memory development	Zhang et al.^184^
	hnRNP A1/Oct4	Mouse	↓ senescent cells	Han et al.^215^
	–	Mouse	↓ senescent cells	Roberts et al.^213^
PUFA-rich diet	–	Mouse	↓ effector memory CD4^+^ T cells	Cucchi et al.^185^
Glucose and carbohydrate restriction	AMPK	Worm	↑ ROS, ↑ oxidative stress resistance response	Schulz et al.^22^
	NHR-49/CBP	Worm with Huntington's disease	↓ proteotoxicity, ↑ lifespan	Marcellino et al.^79^
High-fibre diet/short-chain fatty acids (SCFAs) supplements	FFAR2, FFAR3	Mouse	↑ SCFAs levels, ↑ ILCs proliferation	Sepahi et al.^190^
	–	Mouse	↑ microbial CAZymes activity	Wastyk et al.^201^
Fermented/ probiotic diet	–	Mouse	↑ alpha diversity of the gut microbiota, ↓ inflammation	Wastyk et al.^201^
Protein restriction	TOR	Worm	↓ protein synthesis	Bonawitz et al.^387^
	TOR	Fly	↑ lifespan	Jensen et al.^388^
	–	Fly	↑ lifespan	Lee et al.^389^ Stefana et al.^390^ Fanson et al.^391^
	mTORC1	Mouse	↓ BCAA and glucose metabolism	Solon-Biet et al.^392^
	GCN2-ATF4		↑ FGF21, ↑ food intake, ↑ energy expenditure, ↓ body fat weight, ↑ body lean weight	Laeger et al.^98^
*Amino acid intervention*				
Asparagine+ glutamate restriction	MSN2/4	Yeast	↑ lifespan	Powers et al.^393^
BCAA restriction	mTOR	Mouse	↑ lifespan in males, ↓ frailty	Yu et al.^163^
Cystine restriction	GCN2/ATF4/SESN2/mTOR	Mouse Human	↓ tumour growth, ↑ efficiency of chemotherapy	Wu et al.^351^
Methionine restriction	TOR	Yeast	↑ lifespan	Lee et al.^394^
	–	Yeast	↑ autophagy, ↑ lifespan	Ruckenstuhl et al.^395^
	–	Mouse	↓ senescent cells	Parkhitko et al.^214^
	–	Yeast	↑ lifespan	Sutter et al.^94^
	Sestrin	Fly	↑ lifespan, ↑ regulation of ISCs and gut health, ↓ age-related gut pathology	Lu et al.^396^
	GH	Mouse	↑ lifespan	Brown-Borg et al.^397^
	–	Mouse	↑ macrophage migration inhibition factor in liver, ↓ insulin/ IGF-1, ↓ glucose, ↓ thyroid hormone	Miller et al.^398^
	–	Rat	↑ lifespan	Zimmerman et al.^399^
	–	Rat	↑ lifespan, ↓ mitochondrial ROS, ↓ oxidative damage	Sanz et al.^400^
*Micronutrient intervention*				
K restriction	Vacuolar acidity	Yeast	↑ lifespan	Sasikumar et al.^401^
Fe restriction	Proteostasis	Worm	↑ lifespan	Klang et al.^402^
Zn restriction	DAF-16	Worm	↑ lifespan	Kumar et al.^403^
Zn supplement	BMP4/GPR39	Mice	↑ T cell development	Iovino et al.^183^
Se supplement	GPX4	Mouse Human	↑ follicular helper T cells, ↑ antibody responses for influenza vaccination	Yao et al.^182^
High-salt diet	–	Mouse	↑ anti-tumour function of NK cells, ↓ tumour growth	Rizvi et al.^189^
Metabolite intervention				
NR supplement	–	Overweight or obese female	↑ muscle insulin sensitivity	Yoshino et al.^65^
	Clock repressor PER2	Old mouse	↑ NAD^+^ level, ↓ aging	Levine et al.^66^
	cGAS-STING	APP/PS1 mutant mouse	↑ NAD^+^ level in brain, ↓ inflammatory cytokines, ↑ cognitive and synaptic function	Hou et al.^67^
Spermidine supplement	eIF5A	Mouse	↑ memory B-cell response	Zhang et al.^186^

*AKH* adipokinetic hormone, *AMPK* AMP-activated protein kinase, *ATF4* activating transcription factor 4, *ATF7* activating transcription factor 7, B*CAA* branched-chain amino acids, B*MAL1* brain and muscle Arnt-like protein 1, *BMP4* bone morphogenetic protein 4, *CBP* CREB-binding protein, *cGAS* cyclic GMP-AMP synthase, *DAF-16* abnormal dauer formation 16 (FOXO ortholog), *eIF5A* eukaryotic initiation factor 5A, *EPA* eicosapentaenoic acid, *ER* endoplasmic reticulum, *FA* fatty acid, *FAO* fatty acid oxidation, *FFAR2* free fatty acid receptor 2, *FFAR3* free fatty acid receptor 3, *FGF21* fibroblast growth factor 21, *FOXO1* forkhead box O1, *FOXO3* forkhead box O3, *FOXO4* forkhead box O4, *GCN2* general control nonderepressible 2, *GH* growth hormone, *GPR39* G-protein coupled receptor 39, *GPX4* glutathione peroxidase 4, *GSH* glutathione. *H3K9* histone H3 lysine 9, *hnRNP A1* heterogeneous nuclear ribonucleoprotein A1, *IGF-1* insulin-like growth factor 1, *ILCs* innate lymphoid cells, I*SCs* intestinal stem cells, *LA* linoleic acid, *miR-144* microRNA 144, *MSN2/4* multiple suppressor of SNF1 mutation 2/4, *mTOR* mammalian target of rapamycin, *NAD* nicotinamide adenine dinucleotide, *NADPH* nicotinamide adenine dinucleotide phosphate, *NHR-49* nuclear hormone receptor-49, *NPT1* nicotinate phosphoribosyltransferase 1, *NR* nicotinamide riboside, N*rf2* nuclear factor E2-related factor 2, *Oct4* Octamer-binding transcriptional factor 4, *PER2* Period2, *PGC-1α* peroxisome proliferation-activated receptor coactivator 1 α, *PKA* protein kinase A, *PLA2G7* platelet activating factor acetyl hydrolase, *PPARα* peroxisome proliferator-activated receptor-α, P*UFAs* polyunsaturated fatty acids, *RHEB-1* Ras homologue enriched in brain (RHEB ortholog), *ROS* reactive oxygen species, *SCFAs* short-chain fatty acids, *SESN2* sestrin 2, *SIR2* silent information regulator 2 (SIRT1 ortholog), *SIRT1* sirtuin 1, *SIRT3* sirtuin 3, *SIRT5* sirtuin 5, S*OD2* superoxide dismutase 2, *STING* stimulator of interferon genes, *Tim* Timeless, *TOR* target of rapamycin (mTOR ortholog), *TORC1* target of rapamycin complex 1, *UPR* unfolded protein response.

### Nutrient-mediated mechanisms

#### Metabolic regulators

##### mTORC1

One of the principal nutrient-related molecular mechanisms is the mammalian target of rapamycin (mTOR) kinase. mTORC1 and mTORC2, which are both composed of diverse protein subunits phosphorylating distinct substrates, constitute two separate complexes of mTOR kinases.^7^ In short, mTORC1 controls an extensive range of external stimuli, including the availability of oxygen, insulin/insulin-like growth factor 1 (IGF-1), glucose, amino acids and cholesterol, while mTORC2 acts predominantly as an effector of phosphoinositide 3-kinase (PI3K) signalling.^8^ mTORC1 integrates numerous extracellular and intracellular nutrient signals, which involve autophagy, ribosomal biogenesis and protein translation as well as biogenesis of proteins, lipids and nucleotides. A previous report discussed the regulation of mTORC1 in detail.^7^ Inhibition of mTOR activity via genetic modification and rapamycin treatment extends lifespan in species such as yeast, worms, Drosophila and mice.^7,9^ Subsequently, diet restriction has been found to impair mTOR activity and favourably impact many age-related disorders in humans. For example, calorie-restricted diets enhance longevity, improve cognitive function, reinforce cardiac function, ameliorate metabolic pathogenesis and reduce the incidence of cancer.^10–13^ Likewise, mTOR inhibitors such as rapamycin and perhexiline maleate have been found to mimic the health-promoting effect of CR.^13,14^ However, the wide-scale application of rapamycin has been impeded owing to a number of side effects.^15^ In mice, the ‘off-target’ restraint of mTORC2, which could be interrupted via chronic therapy with rapamycin in vivo, probably mediates these side effects.^16,17^ These findings have provoked great interest in distinguishing feasible dosing regimens for rapamycin and any other drugs specifically targeting mTORC1 that could maximize the beneficial effects and minimize the side effects simultaneously.

##### AMPK

AMP-activated protein kinase (AMPK), as a sensor of mitochondrial stress and nutrient status, can be activated under elevated AMP:ATP ratio conditions, embodying a detector of cellular energy status.^18^ AMPK switches on several catabolic pathways, such as glycolysis and fatty acid oxidation, to supplement ATP levels as a timely response to low levels of cellular energy.^19^ In addition, AMPK regulates many cellular signalling pathways, including mTORC1, by phosphorylating raptor and tuberous sclerosis complex 2 (TSC2).^18^ Both intermittent fasting (IF) and time-constrained feeding have been reported to activate AMPK to improve health and function in multiple species.^20,21^ In *C. elegans*, glucose restriction (GR) stimulates mitohormesis, which is defined as the upregulation of reactive oxygen species (ROS) levels along with the activation of the oxidative stress resistance response. This GR-induced effect depends on AMPK and is essential for its prolonged longevity function.^22,23^ In addition, AMPK activation contributes to longevity in *C. elegans* by sustaining mitochondrial network homoeostasis and functional coordination with peroxisomes to promote fatty acid oxidation.^24^ Importantly, metformin, an agonist of AMPK, has been found to decrease the level of blood glucose, retard tumour progression and reverse cognitive impairment.^13,25^ In summary, the data suggest that AMPK stimulators as dietary adjuvants may favour healthspan.

##### FOXO

Forkhead box O transcription factors (FOXOs) are downstream effectors of the insulin pathway involved in the regulation of stress responses, metabolic homoeostasis, cellular proliferation, and development.^26^ The FOXO family includes FOXO1, FOXO3, FOXO4 and FOXO6, which share a forkhead domain that is an exceedingly conserved DNA-binding domain (DBD) consisting of 100 amino acids.^26^ FOXO activity in response to diverse environmental stimuli is a pivotal regulator in cellular homoeostasis and is chiefly modified via phosphorylation, acetylation, ubiquitylation and methylation.^26^ Crucially, FOXO activity is mainly inhibited by PI3K-AKT signalling but is activated by various cell stresses.^26^ Furthermore, FOXO is regulated by AMPK, sirtuins, and mTOR status.^26^ For example, in mammalian cell cultures, AMPK activation also directly participates in FOXO3 phosphorylation and FOXO3 transcriptional regulation without affecting its nuclear localization.^26^ The role of FOXO is observed in nutritional intervention. CR on peptone dilutions and solid-media growth plates stimulates daf-16/FOXO activity to extend lifespan in *C. elegans.*^27^ In addition, the activation of dFOXO in a fat body of an adult Drosophila alters the response to dietary restriction but is not thought to extend lifespan.^28^ In mice, FOXO1 is increased in liver and skeletal muscles, while FOXO4 is elevated in skeletal muscles and adipose tissue, after CR.^29,30^ FOXO3^+/−^ and FOXO3^−/−^ mice do not live longer after experiencing CR.^31^ In addition, CCAAT/enhancer-binding protein β (C/EBPβ), which is an Aβ and inflammatory cytokine-activated transcription factor, could result in abnormal neural excitation and cognitive dysfunction by selectively triggering inhibitory GABAergic neuronal degeneration through blocking FOXOs. *C. elegans* neurons overexpressing CEBP-2 or LGMN-1 (asparagine endopeptidase) can shorten lifespan and diminish daf-16/FOXO-induced longevity.^32^ Consequently, FOXO has been discovered to support the beneficial effects of CR. Although it is commendably acknowledged that the regulation of CR-mediated lifespan extension critically requires FOXO, we still have no idea how FOXO regulates the gene expression programme that is significant for superior survival during CR. Moreover, identifying factors such as protein partners and posttranslational modifications, which play vital roles in regulating the pattern by which FOXO signalling integrates signals derived from miscellaneous external stimuli in vivo, is crucial for comprehending its function during CR. Further context-specific research concerning the function of FOXO is urgently needed.

##### Sirtuins and NAD

Sirtuins perform multiple catalytic functions, including roles as deacylases, demalonylase, desuccinylase, depalmitoylase, demyristoylase, and mono-ADP-ribosyltransferase.^33^ Levels of the cellular coenzyme nicotinamide adenine dinucleotide (NAD^+^) play a vital role in the deacylase function of sirtuins.^34^ A sirtuin consists of seven subunits (SIRT1-7) with distinct intracellular localizations. SIRT1, SIRT6 and SIRT7 are distributed in cell nuclei; SIRT2 is mostly in the cytoplasm but is transported into the nucleus; and SIRT3, SIRT4 and SIRT5 reside in mitochondria. They have important effects in regulating cellular metabolism, especially in glucose and lipid metabolism.^33^ In yeast, Sir2 (a homologue of mammalian SIRT1) is essential for CR-modulated lifespan.^35,36^ Similarly, Sir2 homologues also participate in the regulation of lifespan in nematodes and flies and significantly influence their responses to dietary limitation.^37^ An accumulating body of research has revealed that CR increases the expression of SIRT1 in several types of tissue in rats and human cell cultures,^38,39^ and humans with CR exhibit upregulated SIRT1 levels in skeletal muscle.^40^ Furthermore, SIRT3 and SIRT5 cause similar alterations in response to CR in mice.^41,42^ Mice overexpressing SIRT1 by genetic modification display a lean and metabolically active phenotype with decreased circulating levels of cholesterol and insulin and improved glucose tolerance.^43^ Nevertheless, mice systemically overexpressing SIRT1 fail to show prolonged lifespan.^44^ The overexpression of SIRT2 extends the lifetime of progeroid mice, which lack BubR1.^45^ Deletion of SIRT3 shortens lifespan,^46^ while elevated SIRT3 levels can enhance haematopoietic stem cell regenerative capacity.^47^ A large amount of evidence shows that obliteration of SIRT6 and SIRT7 induces infirmity and shortens lifespan.^48,49^ However, overexpression of SIRT6 in transgenic male mice can extend healthspan and lifespan;^50^ in addition, lifespan is lengthened in mice with Hutchinson–Gilford progeria syndrome that overexpress SIRT7.^51^ Mechanistically, the transcriptional activity of FOXO is directly regulated by SIRT1 via deacetylation, which could activate FOXO-dependent transcription of genes responsive to stress and prolong lifespan.^52,53^ In mice, the activity of the transcription factor coactivator PGC-1α, considered a key regulator of mitochondrial biogenesis and function, is also deacetylated and regulated by SIRT1.^54^ When AMPK phosphorylates PGC-1α at Thr177 and Ser538, PGC-1α is prone to deacetylation and activation by SIRT1.^55^ SIRT3 plays an essential role in enhancing the mitochondrial glutathione antioxidant defence system in cochlear cells as well as in the neocortex and liver to promote CR-mediated reduction of oxidative damage.^56^ This finding is consistent with a deficiency oxidative metabolism since misregulation of protein acetylation in SIRT3 knockout mice during CR.^57^ SIRT6 enhances DNA stability and represses senescence by facilitating DNA double-strand break repair through poly (ADP-ribose) polymerase 1 (PARP1) activation.^58^ The suppression of long interspersed element class 1 (LINE1) elements via SIRT6, which leads to DNA damage and inflammation, is probably another pivotal mechanism.^59^

Sirtuins are connected with diet as well as with metabolism, owing to their demand for NAD^+^. NAD^+^ functions as not only a master coenzyme in redox reactions but also a cofactor for non-redox NAD^+^-dependent enzymes, including sirtuins and PARPs.^34^ NPT1 (a NAD^+^ synthesis enzyme) and PNC1 (encoding a vital enzyme in the NAD^+^ salvage pathway) are essential for CR-mediated lifespan extension in both yeast and worms.^36,60,61^ NAD^+^ levels decrease with age and age-associated diseases.^35^ Specifically, senescent cells progressively accumulate in liver and visceral white adipose tissue (WAT) with ageing; moreover, they secrete inflammatory cytokines to induce tissue-resident macrophages to proliferate and express the NAD^+^-consuming enzyme CD38. These macrophages reinforce the activity of CD38-dependent NADase, thereby decreasing tissue NAD^+^ levels.^62^ Furthermore, adipose-specific overexpression of nicotinamide phosphoribosyltransferase (NAMPT, an NAD^+^ synthetase), which is the rate-limiting enzyme in a principal NAD^+^ synthesis pathway, was recently reported to elevate NAD^+^ levels in several types of tissue; ameliorate numerous measures of physical performance, metabolic health and cognition; and extend the lifespans of female mice.^63^ Hence, interventions to increase levels of NAD^+^ have prospective therapeutic capacity in ageing and age-related disease.^35^ With regard to this point, dietary intervention is extremely beneficial since it can boost NAD^+^ levels.^64^ Nutritional supplementation with NAD^+^ or NAD^+^ precursors is under active investigation as an approach to promote healthy ageing and intervene in diseases.^35^ For example, in women with prediabetes who are overweight or obese, nicotinamide mononucleotide, an NAD^+^ precursor, has been shown to ameliorate muscle insulin sensitivity, insulin signalling and remodelling.^65^ Similarly, nicotinamide riboside (NR), an NAD^+^ precursor, strikingly reshapes metabolic and stress-response pathways that decline with ageing by inhibiting the clock repressor PER2. NR supplementation in old mice can replenish NAD^+^ to youthful levels that enable improved health.^66^ In APP/PS1 mutant transgenic mice, dietary NR increases brain NAD^+^ levels, reduces the expression of proinflammatory cytokines, and improves cognitive and synaptic functions.^67^

In summary, accumulating studies to date indicate that the sirtuin family probably has a significant function during CR. Current investigations are aimed at defining the targets and enzymatic functions of sirtuins, as well as the character of each sirtuin in the regulation of healthspan, longevity and metabolism.

#### Nutritive metabolism pathways

##### Mineral metabolism

Numerous minerals are universally acknowledged to exert bioactivity through element chelation, giving rise to modulation of antioxidant capacity or microbiome metabolism among other physiological processes. Dietary minerals mainly include Na, K, Cl, P, Fe, Zn, Mg, Se, Cu, I and Ca. The dietary functions of some essential elements in health and disease deserve to be thoroughly clarified.

First, potassium, as the most abundant cation intracellularly, has a close mutual association with sodium. The potassium-sodium gradient between intracellular and extracellular compartments prompts a series of cellular processes that maintain homoeostasis of other metabolites. The potassium balance in mitochondria is a dominant mechanism that regulates mitochondrial redox capacity and charge. Many studies have reported that changes in mitochondrial potassium currents may play substantial roles in the development of neurodegenerative and cardiovascular diseases.^68^

Calcium is essential for living organisms along with normal body function. Calcium is linked with an array of biological events, including immune responses, cell death, cell differentiation, transmission of nerve impulses, muscle contraction, and enzyme activation. Calcium status disorders are conducive to the pathogenesis of bone diseases as well as increased risks of metabolic diseases and epithelial cancer.^69^ Calcium can only be provided with food to satisfy its requirement. Therefore, it is highly necessary to obtain the proper quantity of calcium from the diet for normal body function. Since a low cytosolic calcium level is crucial for cellular function, calcium oscillations are able to act as secondary messengers for diverse stimuli that range from proliferation to apoptosis. Calcium has an extensive array of roles within the mitochondria. Calcium is critically essential for the activities of a few enzymes within the electron-transport chain and tricarboxylic acid (TCA) cycle, including the rate-limiting isocitrate dehydrogenase. It also promotes the conveyance of adenylate and synthesis of ATP within mitochondria, with a parallel elevation in mitochondrial membrane potential.^68^

In addition, magnesium is not only the most abundant divalent intracellular cation in humans but also the second most concentrated intracellular ion after potassium. It has been traditionally considered that magnesium is the cofactor of approximately 300 regulatory enzymes; however, current databases have enumerated over 600 enzymes for which magnesium acts as a cofactor. Magnesium is related to several primary cellular processes, including ATP-dependent biochemical processes, where magnesium is a component of the activated Mg-ATP complex, glucose metabolism, DNA synthesis, RNA expression, blood pressure control, and neural and muscular cell signalling. Magnesium deficiency has also been reported to be linked with oxidative stress, low-grade inflammation, insulin resistance, and metabolic syndrome.^70^

Next, Fe, as an indispensable mineral for sustaining homoeostasis in humans, is critically essential for quite a few cellular reactions, encompassing DNA synthesis, cell division and growth, immune responses, protein metabolism, oxygen transportation through haemoglobin, production of various neurotransmitters, thyroid hormone regulation, oxidation–reduction reactions and erythropoietic functioning within connective tissue. In addition, Fe is also a vital element for numerous enzymes related to metabolic reactions, such as peroxidase and catalase, together with cytochrome. Although one of the main functions of Fe is boosting oxygen diffusion into mitochondria, it might also be adverse owing to its oxidative role in somatic cells. In addition, Fe is absorbed or stored in the form of ferritin in oxidative enzymes. This component is largely found in myoglobin and haemoglobin, and the systemic level is controlled by the balance of its intake, utilization and storage.^71^

Cu, as a trace element pivotal for enzyme function, has a valuable dual function as both a pro-oxidant and an antioxidant. It functions as a catalytic cofactor for an array of enzymes, including Cu/Zn superoxide dismutase (SOD), lysyl oxidase, and ceruloplasmin (CPO), which plays a significant role in the integrity and strength of the heart and blood vessels. Cu is also a crucial requisite for Fe absorption and mitochondrial respiration. Excess copper directly binding to lipoylated constituents in the TCA cycle could further lead to aggregation of lipoylated protein as well as subsequent iron-sulfur cluster protein loss, which would force proteotoxic stress and eventually cell death.^72^ Increased Cu levels are able to enhance ROS production and consequent oxidative stress, causing the oxidation of DNA, proteins, lipids, homocysteine and other molecules. Cu deficiency, on the other hand, can induce peroxidative damage. Moreover, not only Cu deficiency but also Cu overload has crucial effects in atherogenesis.^71^

Zn is the second most common transition metal in humans after Fe. Considering its prevalence in the structure of diverse proteins and enzymes, Zn plays a predominant role in normal cell structure and catalytic function, particularly in the central nervous and immune systems. It is also pivotal to cell growth and division as well as repair, haemostasis, energy-producing functions, wound healing, carbohydrate catabolism, thrombosis, encompassing fibrinolysis, NO synthesis, coagulation and anticoagulation. In addition, intracellular Zn plays a vital role in redox signalling pathways and contributes to antiapoptotic, antioxidant and anti-inflammatory activities. However, Zn deficiency can result in the oxidation and degradation of essential proteins such as protein kinase C (PKC), the production of C-reactive protein (CRP) and inflammatory cytokines, and ingestion of particles by monocytes and macrophages. Moreover, Zn deficiency can affect the development of diverse organs, including the brain, heart, lung, kidney and skeleton.^71^

Ultimately, selenium (Se), as a micronutrient indispensable for the human body, performs its roles as a component of the amino acid selenocysteine (Sec) which is considered the 21st amino acid in selenoproteins. Se exhibits various significant activities, including antioxidant, immunomodulatory, thyroid metabolism and human fertility effects. Inserted into mammalian selenoproteins, Se is different from other minerals interacting with proteins as cofactors. Throughout the mammalian body, selenoprotein genes have already been validated; however, functions have only been described for half of them. Via a mechanism involving the recoding of the stop codon UGA during translation, Sec is generally located at the active site of an enzyme. The 3′ UTR of the Sec incorporation sequence (SECIS) region is where Sec is incorporated into a protein. The majority of selenoproteins are associated with multiple biological reactions concerning the regulation of the redox state and antioxidant function. One of the well-characterized functions of selenoproteins is redox activity, which is mostly due to three isoforms of thioredoxin reductases and deiodinases as well as five members of the glutathione peroxidase (GPX) family. Se is also crucial for modulating not only inflammatory responses, since it can attenuate the activation of the nuclear factor (NF)-κB pathway, but also thyroid function, on account of the action of deiodinases in the conversion of T4 into its active form T3. Furthermore, Se is critically vital for neurological function, since it can protect the brain from oxidative damage, along with male fertility and reproduction.^73^

In summary, conflicting evidence remains regarding mineral interventions for improving health. Clinical studies of mineral patterns, quantity, and bioactivity in diet must be performed to fully understand the role of mineral metabolism in health and disease.

##### Lipid metabolism

Dietary lipids play a conspicuous role in mental and behavioural health. Dietary fatty acids, as structural building barriers of diverse membranes, are connected with pro- and anti-inflammation mediators, making them infinitely important for human health, growth, development and preservation. The quantity and quality of dietary fat has undergone a vast alteration over the past 10,000 years. A switch from a diet abundant in omega-3 polyunsaturated fatty acids (ω−3 PUFAs) towards a Western diet almost deficient in ω−3 PUFAs but increasing levels of saturated fatty acids (SFAs), trans fatty acids (TFAs) and ω−6 PUFAs results from this alteration accompanying the industrial revolution.^74^ Accordingly, balanced quantities and proportions of dietary lipids may be critical to maintaining health. First, accumulating research on nutrient restriction in mice and flies by tracers reveals an upregulation in both the synthesis and catabolism of fat, showing a shift towards superior lipid utilization.^75,76^ Inhibition of fatty acid synthesis by blocking acetyl-CoA carboxylase (ACC) or mitochondrial β-oxidation leads to a failure of dietary restriction-mediated lifespan extension.^75^ Studies in SIRT1^+/−^ cell culture and mice suggest that SIRT1 contributes to fat mobilization in white adipocytes by blocking the transcriptional effects of the fat regulator peroxisome proliferator-activated receptor-γ (PPARγ).^77^ AMPK is also reported to regulate lipid metabolism by phosphorylating ACC.^78^ In worms, it is critically essential for dietary restriction-mediated longevity and amelioration of proteotoxic effects in polyQ Huntington models to suppress genes controlling fatty acid synthesis, oxidation, and desaturation, such as the nuclear hormone receptors NHR-49/PPARα and NHR-80/HNF4.^79^ Importantly, alternate-day fasting (ADF) is reported to reduce fat mass and cholesterol levels but increase the levels of diverse fatty acids and their catabolite β-hydroxybutyrate in a clinical trial.^80^ Furthermore, a ketogenic diet (KD) has been found to be safe and has the potential to extend healthspan or improve health. The KD boosts fatty acid β-oxidation in the liver to generate ketone bodies, including acetoacetate, acetone, and β-hydroxybutyrate. Ketones are transferred into the bloodstream to various tissues, where they are transformed to acetyl-CoA to fuel the TCA cycle.^81^ In terms of types of fatty acids, the elevated abundance of the mono- and poly-unsaturated fatty acids in response to CR is a key component of cell membrane structures and has been revealed to upregulate pro-survival mechanisms such as cellular detoxification.^82^ Taken together, these results indicate that reinforced rates of both fatty acid synthesis and breakdown are vital controllers of CR-mediated longevity. In addition, special attention should be given when assessing the influence of CR on fatty acid metabolism.

##### Proteostasis

Many proteins and amino acids play a crucial role in sustaining health.^83^ Some specific proteins are detrimental to healthspan, since inhibiting these proteins has been found to extend lifespan in *C. elegans.*^83^ Protein interventions hold the capacity to prevent age-associated insoluble proteins from intracellular accumulation, but an increasing number of studies have shown that CR enables the balance of protein synthesis and degradation. First, CR is reported to stimulate endoplasmic reticulum stress and expedite proteostasis, which contributes to prolonging longevity in *C. elegans.*^84^ Proteostasis inevitably suppresses unfolded protein synthesis and opportunistically degrades these toxic proteins, which requires responses to unfolded proteins, including autophagy and ER stress responses.^85,86^ Key autophagic genes have been found to be indispensable for CR-mediated benefits in healthspan improvement from yeast to humans.^87,88^ For example, intermittent time-restricted feeding (iTRF) mediates lifespan extension in Drosophila, which depends on circadian regulation and autophagy. Autophagic activation at night has been found to be sufficient and essential for the benefit of CR for longevity.^87^ Similarly, mammalian MON2 (a Golgi protein) is upregulated in long-lived *C. elegans*, and MON2 is essential for stimulating autophagic flux by activating the Atg8 orthologue GABARAP/LGG-1 in *C. elegans* to extend longevity.^89^ Starvation, lipid restriction, proteins or special amino acid deletions activate autophagy.^90–92^ Overactive autophagy in response to fasting is partially regulated by activation of AMPK and SIRT1 activity and inhibition of mTOR activity.^13^ The autophagic response is also implemented to mobilize stored lipids via lipophagy to coordinate the metabolic response to food availability and CR.^91^ Moreover, total protein restriction or lack of specific amino acids, such as branched-chain amino acids (BCAAs), methionine, cystine and glutamine, are thought to stimulate the autophagic/lysosomal response, partially depending on mTOR suppression and general control nonderepressible 2 (GCN2) activation.^93–96^ For example, fasting drives lysosomal export of cystine in the fat bodies of Drosophila; subsequently, cystine is transformed to cysteine and metabolized to acetyl-CoA by promoting CoA metabolism. This process limits TORC1 reactivation to maintain autophagy.^92^ As an evolutionarily conserved serine/threonine kinase, GCN2 is able to sense changes in amino acid levels to modulate diverse nutrient-response pathways. For example, GCN2 coordinates inflammation and integrated stress responses to control malignant growth and immune homoeostasis. Ribosome stalling and elevated levels of uncharged tRNAs can activate GCN2, and activated GCN2 induces the phosphorylation of eukaryotic translation initiation factor 2 (eIF2), subsequently selectively stimulating ATF4-mediated translation but blocking translation of most mRNAs.^97^ Fibroblast growth factor 21 (FGF21) secretion in response to acute protein restriction partially depends on the GCN2-ATF4 pathways, whereas chronic protein restriction is capable of directly stimulating ATF4-induced FGF21 release in a GCN2-independent manner.^98^ The sophisticated effects of diet interventions on proteostasis deserve to be elucidated in detail in the context of different strategies and in different tissues.

##### Mitochondrial function

The centre of many metabolic processes is mitochondrial function. Mitochondria are metabolic centres that constructively respond to diet through mitochondrial quality control and alteration of mitochondrial function.^99,100^ Likewise, mitochondrial dysfunction underlies various age-associated diseases.^99^ However, the effects of CR on the improvement of mitochondrial homoeostasis and mitochondrial function are inconsistent. One potential mechanism is that CR reduces mitochondria-generated ROS and ROS-induced damage.^101^ Compared to hepatocellular mitochondria from rats fed a normal diet, those isolated from rats fed a 40% CR diet were found to exhibit reductions in membrane potential, oxygen consumption and ROS production while maintaining ATP generation, which demonstrated that CR was able to augment mitochondrial efficiency.^102^ In the skeletal muscle of humans, short-term CR has no effect on the function of mitochondrial enzymes but facilitates mitochondrial biogenesis to decrease oxidative stress, suggesting that CR is capable of inducing the formation of highly efficient mitochondria.^40^ However, chronic CR fails to boost mitochondrial richness but optimizes oxidative damage to DNA and protein through augmentation of antioxidant scavengers and reduction of oxidant emission.^103^ Similarly, deficiency in mitochondrial fitness, proteotoxicity and mitonuclear protein imbalance can trigger mitochondrial stress, which results in mitophagy and the mitochondrial unfolded protein response (mtUPR) to maintain mitochondrial homoeostasis.^104,105^ In worms and long-lived Snell dwarf mice, the mtUPR can sustain mitochondrial protein stoichiometry and alter electron-transport chain components to prolong lifespan.^106,107^ Incremental studies have also indicated that CR prevents the impairment of mitochondrial DNA (mtDNA).^108^ In addition, fasting or CR induces mitophagy, suggesting enhanced turnover of damaged mitochondria.^109^ Interestingly, the TCA cycle, a main metabolic pathway in the mitochondria, is a centre of cellular energy and metabolism through the compact connection of glucose, amino acids and lipid oxygenolysis. α-Ketoglutarate (αKG) is an intermediate in the TCA cycle, and αKG supplementation has the potential to inhibit the ageing process and prolong healthspan (reviewed in detail in ref. ^110^) Taken together, it is evident that mitochondrial functionality is indispensable for CR-modulated health. However, this evidence is necessary to decipher how dietary interventions affect mitochondrial homoeostasis across species and within various tissues under the circumstances of disease and health.

#### Epigenetic mechanisms

Epigenetics is defined as regulation of gene expression through frequent alteration of the expression of genetic material and DNA configuration in the absence of sequence mutations. Epigenetic alterations are moderate and stepwise but could possibly be rejuvenated. Epigenetics deals with three main types of modulations: (1) DNA methylation, (2) histone modification and (3) ncRNA-mediated gene expression. Indeed, nutritional interventions impact the methylation of DNA, histone modification and ncRNA expression.^111,112^

First, DNA methylation constitutes covalent modifications in CPG dinucleotides, which show a pair of cytosines at the 5-carbon position of CG dinucleotides. DNA methylation enzymes include DNA methyltransferase 1 (DNMT1), DNMT2, DNMT3a and DNMT3b. Folate metabolism preserves balanced quantities of deoxyribonucleic acid and is thus crucial to DNA replication. As a cofactor for enzymes, folate is essential for synthesizing nucleotides and thymidylate.^113^ Similarly, one-carbon units are mainly derived from serine during the homocysteine-methionine conversion process; furthermore, the conversion of methionine to S-adenosyl methionine (SAM) requires the consumption of abundant ATP that can be produced within the de novo synthesis of serine. Liver kinase B1 (LKB1) loss augments DNA methylation through activation of de novo serine biosynthesis.^114^ Serine hydroxymethyltransferase 2 (SHMT2), a key enzyme of serine biosynthesis in mitochondria, has been observed to promote histone and DNA methylation by increasing SAM synthesis.^115^ In addition, an enhancement of αKG levels has been found to lead to the differentiation of tumour cells in a 5-hydroxymethylcytosine (5hmC)-dependent manner.^116^ Methyl donor SAM replenishment is derived from dietetic methionine, serine, vitamins B2, B6 and B12, and folate. A lack of these can weaken DNA methylation, potentially extending lifespan and improving health.^115,117,118^

Histone proteins include eight proteins containing histones H2A, H2B, H3 and H4 in complex octameric structures. As a histone linker, histone H1 connects DNA couplers with nucleosomes. Histone modifications, including methylation, acetylation, phosphorylation, succinylation, hydroxybutyrylation and lactylation, may undergo changes during metabolic events.^119^ For example, CR restricts SAM availability to suppress histone H3K4 methyltransferase SET-2 activity, which leads to activate TFEB/FOXA-mediated autophagy to prolong lifespan.^120^ Meanwhile, high levels of αKG are thought to sustain trimethyl H3K27 and H3K9, and αKG supplementation ameliorates age-related osteoporosis.^121^ Moreover, the histone acetyltransferase p300 enhances the expression of senescence genes. Mechanistically, p300 contributes to the formation of activatory enhancer elements within noncoding regions and dynamic hyperacetylation of chromatin.^122^ Conversely, CR-activated sirtuins and p300 inhibitors reduce the overall level of histone acetylation to improve health.^123–125^ Finally, succinyl-CoA derived from the TCA cycle is the direct donor for succinylation. Lysine acetyl transferase 2A (KAT2A) was first identified as a succinyltransferase and catalyses the succinylation of histone H3 at lysine 79 by binding to succinyl-CoA, which profoundly promotes gene expression.^126^ KAT2A performs protein succinylation, while SIRT7 is endowed with the capacity to act as a histone desuccinylase when DNA is damaged.^127^

Noncoding RNAs (ncRNAs) include long noncoding RNAs (lncRNAs), microRNAs (miRNAs), circular RNAs (circRNAs) and packaging RNAs (pRNAs), and account for a large proportion of the human genome. The majority of ncRNAs fail to encode proteins but exert pivotal effects on translation, mRNA stability and transcription.^128^ As well-established ncRNAs, miRNAs are thought to mediate nutrient-sensing pathways.^129^ For example, some miRNAs have been discovered to regulate metabolic signalling, such as mTOR, AMPK, sirtuins and insulin/IGF-1, by targeting their mRNAs.^130,131^ Likewise, some gene signatures based on diet-mediated miRNA expression can predict health improvement.^132,133^ In addition, miRNAs are capable of being released into body fluids and plasma either in a vesicle-free form or packaged in extracellular vesicles, highlighting that these marked miRNAs could be utilized as non-invasive diagnostic tools and predictors of health and diet-associated diseases.^134,135^ Ultimately, dietary miRNAs originating from plants and milk biologically affect the health of the host by directly targeting host genes and indirectly affecting the function of the microbiome, leading to the development of novel methods to treat or prevent diseases.^136^

Polyphenols are abundant in vegetables and fruits, and possess polyhydroxy phenols as a fundamental structure. They comprise ten subgroups, including acetophenones, lignins, xanthones, benzoquinones, flavonoids and phenolic compounds, based on their chemical structures and properties.^13^ Dietary polyphenols are implemented to prevent certain diseases through diverse mechanisms, such as silencing genes and epigenetic modifications.^13,137–141^ For example, polyphenols function to induce histone modifications such as deacetylation and methylation and inhibit DNA methyltransferase for health improvement. Polyphenols exhibit properties of fundamental alteration of neoplastic or systemic epigenomes and are of great interest for disease prevention and health improvement.

#### Circadian clock modulators

As an inherent punctual system, the circadian clock has the capacity to sustain an all-day rhythmic cycle of physiology, behaviour and metabolism.^142^ Clock genes have rhythmic activity to regulate the transcription of numerous genes in a cyclic way, which in turn synchronizes diverse physiological processes to external stimuli, such as photostimulation. Under this condition, dysfunction of circadian rhythms plays an important role in health.^143^ For example, Reg3γ, a C-type lectin antimicrobial peptide in host, connects intestinal circadian clock to ileal microbes. Fat-enriched diet results in a continuous expression of Reg3γ to drive microbial oscillators.^144^ Apart from light, clock genes can also respond to cues from diet. In mouse and fly models, CR, especially at night, has been found to enhance the amplitude of clock gene mRNA expression to promote longevity.^145,146^ Without the primary clock genes period (Per) and timeless (Tim), flies or mice fail to extend their healthspan even upon TRF.^145,146^ In clinic, it is widely accepted that TRF ameliorates metabolic health. Compared with mid-day TRF (mTRF, food intake restricted to the middle of the day), early TRF (eTRF, food intake restricted to the early part of the day) is reported to be more conducive to enhancing insulin sensitivity, increasing gut microbial diversity, ameliorating inflammation, and reducing body weight, adiposity and fasting glucose.^147^ Mechanistically, circadian rhythms have been found to mediate brain and muscle Arnt-like protein 1 (BMAL1) in the brain and muscle to drive daily fluctuations in NAD^+^ levels and shape the light cycle.^148^ Furthermore, SIRT1 and AMPK interact with these circadian factors and modulate their activity.^142,148,149^ In addition, both autophagy and circadian regulation are necessary when iTRF extends fly longevity and decreases the profile of age-associated biomarkers in the gut and muscle. On an AL diet, autophagic induction only at night is sufficient to prolong lifespan.^87^ Conversely, night-time eating results in misalignment between central and peripheral (glucose) endogenous circadian rhythms and impairs glucose tolerance to increase diabetes risk.^150^ Moreover, deficiency of the specific circadian clock regulators Tim and Per has been reported to lead to changes in cellular respiration through an increase in the uncoupling protein UCP4C in the gut and eventually contributes to prolonging the lifespan of male Drosophila.^151^ How nutrient signals affect circadian clock genes is worth investigating. To identify the metabolic functions of the circadian clock, the integration of multi-omics, including metabolomic and sequential circadian transcriptomic analyses, in many human tissue types is indispensable. Seeking the circadian rhythm-modulated pathways and the potential mechanisms by which they affect health is entirely relevant for individualized translational applications.

### Diet-responsive effectors

#### The diet-endocrine axis

Nutritional complementarity is primary to an organism’s growth and development via regulation of multiple cytokines (leptin, adiponectin, asprosin, acyl-CoA-binding protein and so on) and growth factors and hormones (insulin/IGF-1, FGF21, thyroid-stimulating hormone, prolactin, corticosterone, etc.).^80,152–156^ Insulin and IGF-1 were first discovered to be connected to diet and health. The orthologue of the mammalian insulin/IGF-1 receptor in *C. elegans* is encoded by the daf-2 gene, and its mutation has been found to dramatically prolong longevity.^3^ As in *C. elegans*, relative mutations in genes in the insulin/IGF-1 pathway, such as chico (the insulin receptor substrate-like signalling protein gene),^157^ and mutations in the insulin-like receptor gene (InR),^158^ are observed to prolong lifespan in flies. In mice, knockout of insulin receptor substrate 1 (IRS1) or IRS2 heterozygosity throughout the body and selective deletion of the insulin receptor in fat tissue are capable of extending lifespan.^93^ Similarly, CR or caloric restriction mimetics (CRMs) have been reported to decrease the circulating levels of insulin and IGF-1, which has been regarded as a pivotal longevity mechanism of CR.^13,90,159^ Mediators of CR, such as mTOR, FOXO and SIRTs, are involved in insulin/IGF-1 signalling.^160^ Generally speaking, it is likely that the favourable effects of CR partially depend on the inhibition of the insulin/IGF-1 pathway, and these dietary strategies or drugs for suppressing this signalling may have the potential to improve healthspan in translational research. Nevertheless, IGF-1 is also secreted by Lepr^+^ mesenchymal cells surrounding intestinal crypts, and stimulates local intestinal stem cells (ISCs) functionality and proliferation via binding to epithelial IGF1R.^161^ Moreover, FGF21 is a stress-inducible hormone involved in multiple pivotal metabolic signalling pathways.^162^ Both total protein restriction and particular amino acid restriction stimulate FGF21 secretion in the liver and adipose tissue to augment energy expenditure and improve insulin sensitivity.^163,164^ In responses to protein restriction, FGF21 plays an important role in diverse species, including humans. Long-term protein restriction stimulates the release of FGF21 in hepatocytes and elevates circulatory levels in male C57BL/6J mice and Sprague Dawley rats.^163,165,166^ In the clinic, protein restriction (4–6 weeks) can elevate circulating FGF21 levels.^163,165^ Time-restricted fasting also potently promotes FGF21 release, and transgenic FGF21 expression and recombinant FGF21 treatment have the capacity to improve insulin sensitivity and glucose tolerance in mice.^162^ Interestingly, transgenic overproduction of FGF21 has no effect on food intake or the mTORC1 pathway but independently prolongs healthspan in mice.^167^ Altogether, continuing studies are required to further decipher the specific mechanism by which CR and protein restriction stimulate FGF21 secretion, and these dietary treatments and FGF21 mimics are implemented to sustain health and treat metabolic diseases.

#### The diet-immune axis

Diet has been discovered to impact the immune system (has been reviewed in ref. ^168^) The immune system uniquely contains various cell subpopulations and multiple layers with particular and complementary effectors. In response to external stimuli, including cancer and infection, the interdependent immune network endows the host with the capacity to protect against these various stimuli.^169,170^ Confronted with these challenges, every organ exhibits tissue-specific immune responses, which are mainly reflected in resident immune cells that boost tissue repair and rapid local protection. These immune cells obviously adapt to the regional environment and show metabolic characteristics analogous to those of resident tissue.^171,172^ In parallel, several immune cell subsets and multitudinous immunological factors dynamically exist in circulation and migrate throughout the body to conduct immunosurveillance. When at rest, these immune cells commonly need few nutrients.^173^ However, immune cells targeting dangerous stimuli definitely require heavy consumption of nutrients and energy for functional activation and enormous expansion.^174^ Therefore, the immune system exhibits extreme adaptation and high resilience to thrive under fluctuating nutrient conditions.

The quantity and composition of the diet directly affects the immunological response by modulating nutrient availability. Fluctuations in nutrients and/or reduced dietary intake hold the potential to favour optimal immune responses. Recently, some studies have demonstrated that CR or IF can moderate the harmful off-target effects of allergies and autoimmunity and protect against cancer and pathogens through optimization of immune responses.^175–179^ In *C. elegans*, CR or reduced insulin/IGF-1 signalling has been found to downregulate p38-ATF7 signalling to a basal level in the innate immunity pathway, which contributes to the extension of lifespan.^178^ In mice and humans, CR ameliorates ageing-associated alterations in transcriptional regulatory networks, expression of key genes and the composition of cell types. The proinflammatory type of immune cells has been conjectured to contribute to ageing, while CR beneficially disrupts age-induced eccentric interactions within immune cells, similar to the excessiveness of proinflammatory ligand–receptor combinations.^179^ In healthy humans, a 2-year CR inhibits platelet-activating factor acetyl hydrolase (PAF-AH) in the adipose tissue surrounding the thymus, inactivates the NLRP3 inflammasome, prevents age-associated inflammation, and reduces thymic lipoatrophy to improve metabolic health.^100^ In terms of dietary constituents, ions are crucial in immune system homoeostasis. An excessive sodium chloride diet not only motivates maturation of DCs and activates their antigen-presenting capacity but also stimulates the generation of proinflammatory factors and autoantibodies, which leads to lymphadenectasis and splenomegaly, and exacerbates renal diseases.^180^ Likewise, the potassium channel K2P18.1 mediates the development and function of Tregs by facilitating FOXP3 expression. Mechanistically, K2P18.1 forcibly drives continuous Ca2^+^ influx to increase FOXP3 transcription in an NFAT- and NF-κB-dependent manner. The K2P18.1 activator nitroxoline is detrimental for urinary tract infections via a rapid and reversible increases in Tregs.^181^ Moreover, Se supplementation has been found to enhance GPX4 expression in T cells, increase the numbers of follicular helper T cells and promote antibody responses in mice and humans after influenza vaccination.^182^ Similarly, zinc as a dietary supplement stimulates the secretion of bone morphogenetic protein 4 (BMP4) in thymic endothelial cells by binding to its receptor GPR39 to promote T-cell development.^183^ In lipid-associated metabolism, ketogenesis-derived β-hydroxybutyrate enables epigenetic modification of Lys 9 of histone H3 (H3K9) and hydroxybutyrylates Ppargc1a (which encodes PGC-1α) and Foxo1, upregulating Pck1 expression to enhance the pentose phosphate and glycogen pathways in CD8^+^ memory T cells.^184^ In addition, an ω−3 PUFA-rich diet has been found to decrease the quantity of effector memory CD4^+^ T cells in fat tissues and alter lipid profiles in plasma, adipose tissues and lymphoid organs. Docosahexaenoic acid (DHA) and eicosapentaenoic acid (EPA) treatments interfere with cytoskeletal rearrangements to decrease the motility of CD4^+^ T cells and recede their polarity.^185^ Other small-molecule metabolites in the diet also influence the differentiation and function of immune cells. Supplementation with spermidine, an endogenous polyamine metabolite, reinstates memory B-cell responses. Specifically, spermidine stimulates the hypusination of eukaryotic initiation factor 5A (eIF5A), further activating TFEB-dependent autophagy.^186^

Nutrients mediate the function and composition of the microbiota to indirectly coordinate host immune responses. First, fat-enriched diet is reported to perturb the diversity of gut microbiome and repress the expression of major histocompatibility complex class II (MHC class II) in ISCs, which impairs immunosurveillance for tumorigenesis.^187^ Similar to high-fat diet, high salt intake is found to particularly deplete *Lactobacillus murinus* in mice, enrich Th17 cells and increase blood pressure.^188^ However, a high-salt diet augments gut permeability and increases the gut and intratumor richness of Bifidobacterium, which results in tumour regression by activating the antitumor function of NK cells.^189^ In addition, dietary fibres can be decomposed into short-chain fatty acids (SCFAs). These SCFAs optimize the proliferation of innate lymphoid cells (ILCs) in the intestines. For example, SCFAs stimulate expansion of ILC2 cells by binding to their receptors, such as free fatty acid receptor 2 (FFAR2) and FFAR3. However, SCFAs have the capacity to inhibit the proliferation of ILC2s in an FFAR-independent manner.^190^ Taken together, alimentative treatments, including CR and compositional alteration, optimize and educate the immune response in an evolutionarily conserved manner.

#### The diet-gut axis

Recently, diet-modulated gut microbiomes and their metabolites have exerted prominent roles in shaping health.^191^ Considering that diet-induced changes significantly affect the gut microbiome, CR has the potential to exert an immense effect.^192^ CR markedly alters the composition and function of gut microbiota to stimulate beige fat and improve metabolic state. Mechanistically, CR has been found to reduce lipid A biosynthesis through diminution of bacterial enzymes, further recruit alternative activated macrophages and eosinophils in adipose tissue to ameliorate diet-induced fatty liver and promote the development of beige fat.^193^ Meanwhile, CR enables to reprogramme the microbial bile acid metabolism to stimulate brown adipose.^194^ However, a very-low-calorie diet severely impairs bacterial abundance and restructures the gut microbiome. Specifically, this dietary strategy has been found to result in an enrichment in *Clostridioides* difficile that was related to weight loss and metabolic improvement in a toxin-dependent manner.^192^ In addition, *Lactobacillus gasseri* located in upper small intestine is found to upregulate the expression of long-chain acyl-CoA synthetase 3 (ACSL3), which serves as a pre-absorptive mediator to regulate systemic glucose homoeostasis.^195^ Clinically, fasting influences the generation of SCFAs by altering bacterial taxa and related genes in the gut. The profiles of immune cells, such as Treg, Th17 cells and CD8^+^ effector T cells, and microbial taxa, including *Akkermansia*, *Ruminococcaceae*, *Desulfovibrionaceae* and *Hydrogenoanaerobacterium*, have been speculated to control systolic blood pressure in response to fasting.^196^ Homogeneously, CR re-establishes a favourable gut microbiome, contributed to higher overall alpha diversity and augments the proportions of naïve B and T cells to delay immunosenescence.^197^ Recent evidence in clinical indicates that the low-energy diet (LED) significantly increases the richness of *Akkermansia* and *Christensenellaceae* R-7 associated with metabolic improvements in overweight and prediabetic individuals.^198^ Inconsistently, other clinical results have indicated that the gut microbiome remains highly individual-specific but stable in response to CR.^11^ In addition, dietary contents impact microbial community stability.^199^ For example, ketone bodies reduces the levels of proinflammatory Th17 cells in the gut by electively suppressing bifidobacterial growth.^200^ In contrast, a high-fibre diet has no effect on microbial community diversity but increased microbiome-encoded glycan-degrading carbohydrate active enzymes (CAZymes), whereas a diet enriched in fermented food steadily decreases inflammatory markers and increases microbiota diversity.^201^ Dietary fibres are universally considered to be beneficial for our health via the gut microbiome.^202–204^ For example, a recent study with multiomic signatures shows that arabinoxylan consumption is capable to reduce cholesterol level mainly depending on a reduction in low-density lipoprotein (LDL) and an increase in bile acids. By contrast, long-chain inulin results in liver inflammation and upregulation of alanine aminotransferase.^203^ One of potential mechanisms is that the different structures of dietary fibres contribute to the variants of gut microbiome and their metabolic functionality.^204^ However, dietary fibre deficiency has been found to expedite the utilization of glycoproteins by mucus-eroding microbiota, such as *Citrobacter rodentium*, eventually leading to intestinal barrier dysfunction and lethal colitis.^205^ Dietary thymidine and serine alter microbial composition via different mechanisms. The microbial conversion of 5-fluoro 2'deoxyuridine (FUdR) into toxic 5-fluorouridine-5'-monophosphate (FUMP) in *C. elegans* can be accelerated by thymidine supplementation. In contrast, serine alters one-carbon metabolism to exacerbate DNA toxicity in *E. coli.*^206^ Ultimately, the gut microbiota plays a crucial role in the interaction between host circadian rhythms and dietary timing (which has been reviewed in refs. ^207,208^) For instance, dietary ingredients and rhythmicity modulate the small intestine microbiome. Changes in feeding contents or time have been found to disrupt the circadian clock, resulting in barrier disruption with extensive import of microbial products to drive Crohn-like enteritis.^209^

#### The diet-senescence axis

Cellular senescence has been identified as a permanent arrest of cell proliferation in response to several stressful stimuli, such as DNA damage, oxidative stress, telomere dysfunction, oncogene activation and metabolic dysfunction.^210^ Accumulation of senescent cells in tissues contributes to organismal ageing and age-associated diseases and impairs tissue regeneration via diverse secretory factors and metabolites.^211^ Various nutritive therapies have been found to eliminate these senescent cells. CR holds the capacity to decrease the markers of senescent cells and relieve age-related inflammation in both mouse and human tissues.^212^ These mechanisms reinforce DNA repair, inactivation of oncogenes, reduced ROS production and clearance of damaged mitochondria by mitophagy (which has been reviewed previously^210^) Other dietary interventions, such as KD^213^ and methionine or serine restriction,^115,214^ can similarly eliminate senescent cells. β-hydroxybutyrate generated by KD decreased the level of senescence biomarkers in endothelial cells and vascular smooth muscle.^215^ In comparison, methionine restriction reduced senescent biomarkers by enhancing methylation and promoting one-carbon metabolism.^216^ Studies have demonstrated that appropriate nutritional treatments can deplete senescent cells to shape a supporting metabolic state.

#### The diet-nerve axis

The contribution of the nervous system to food intake and preferences is pivotal. Several landmark reports have shown that the central nervous system (CNS) maintains systemic metabolic homoeostasis in response to nutrition. For example, Capa, a homologue of mammalian neuromedin U, is released from a complex of six neurosecretory cells in the CNS of Drosophila in response to circulating levels of nutrients. Capa is capable of controlling energy homoeostasis via combination with its receptor (CapaR) in peripheral tissues to sustain energy homoeostasis.^217^ Similar to the CNS, the peripheral nervous system (PNS) also possesses nutritional sensors. For instance, neuropod cells in the intestine are indispensable for the preference for nutritive sugars over non-caloric sweeteners via glutamatergic signalling transduction.^218^ Enteroendocrine cells, as sensors of alimental amino acids in the gut, secrete diuretic hormone 31 (DH31). DH31 binds to the DH31 receptor (DH31R) in brain neurons and excites courtship behaviour.^219^ Importantly, nutritional intervention profoundly impacts the functionality and homoeostasis of the nervous system. Age-associated cognitive decline is reported to be delayed by CR in complex mammals, including humans.^220^ One potential possibility is that CR can promote neuron survival and enhance neurogenesis.^221^ Less cell proliferation and fewer progenitor cells and neuroblasts observed in sites of neurogenesis in the subgranular layer (SGL) and dentate gyrus subventricular zone (SVZ) are viewed as classical structural alterations induced by ageing.^222^ Interestingly, alternate-day CR increases cell survival and induces neurogenesis in the dentate gyrus in 8-week-old mice.^223^ Moreover, CR is discovered to elevate the levels of neurotrophi ω−3 and brain-derived neurotrophic factor.^223^ Similarly, an increased proliferation of progenitor cells and neural stem cells in the hippocampus is only observed in young mice undergoing 40% CR.^224^ It is non-negligible that mice with both CR and calorie dilution (CD) exhibit a prominent elevation of starvation-related genes in hypothalamus.^225^ In addition, composition-specific diet influences neurologic function. A diet with reduced dietary protein intake or rapamycin treatment reduces food intake in mice, but these mice do not appear to be hyperphagic when returned to a protein-rich diet. A very low-protein diet caused weight decline and improved glucose tolerance partially through hypothalamic mTOR signalling.^226^ Then, KD is found to improve cognition in mild cognitive impairment,^227^ while methionine restriction increases FGF21 expression in serum, liver, and brain to alleviate age-associated cognitive decline.^228^ Interestingly, the supplement with non-essential amino acids results in appetitive suppression through direct activation of hypothalamic orexin/hypocretin neurons.^229^

Although the regulation of nutrient-sensitive mechanisms and their modulators by nutrimental intervention is well established, subsequent studies can concentrate on identifying and validating the polymorphic functions, which will require translational studies to develop individualized therapeutic regimens for disease treatment and healthspan improvement.

## Dietary intervention in tissue health and disease

**Fig. 3:**
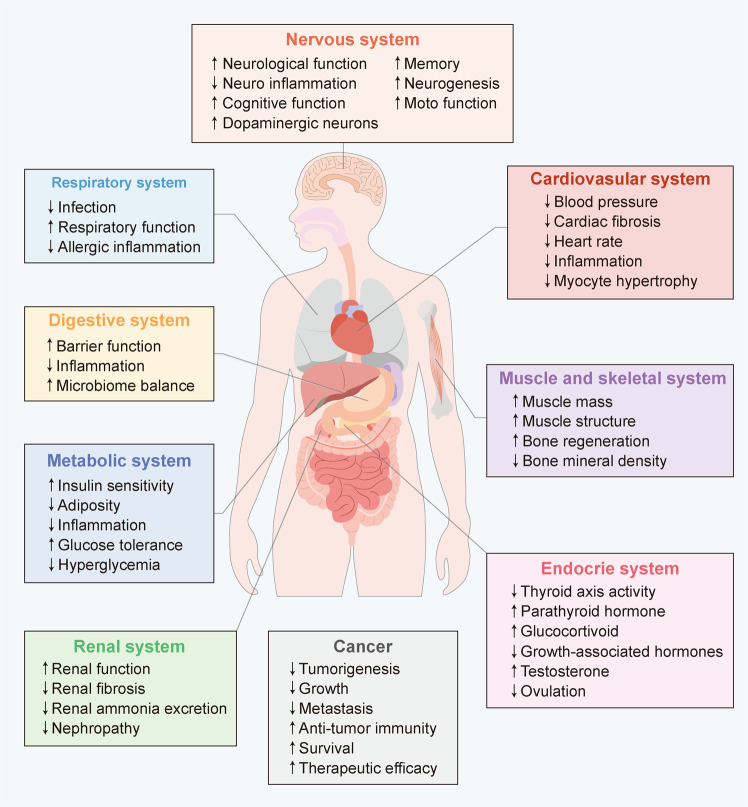
Functionality of dietary interventions on human tissues under health and diverse disease states. Dietary interventions exert beneficial effects across varying aspects, including nervous system, cardiovascular system, respiratory system, metabolic system, renal system, endocrine system, and digestive system as well as cancer

Considering that diet-mediated signalling exists throughout the entire body, diet-derived effects on health and disease have been assumed to occur in all tissues and organs. Consequently, nutrients influence tissue to varying degrees and in diverse ways (Fig. 3). Furthermore, multiple nutritional interventions have been demonstrated to broadly relieve the disease process and immensely strengthen the therapeutic effect (Table 3). In addition, we also summarize the current clinical trials related to dietary interventions (Table 4). Thus, we elaborate the mechanisms by which diet impacts health and diseases, which can aid in the development of precision-nutrition therapeutics.

**Table 3:** The roles of dietary interventions in disease models

Disease	Regimen	Model	Effect	Reference
Metabolic syndrome	CR	Mouse	↑ M2 macrophage, ↑ eosinophils, ↑ fat beige	Fabbiano et al.^234^
		old mouse	↑ adiponectin	Miller et al.^235^
		db/db mouse	↑ insulin sensitivity, ↑ β cell mass, ↑ utilization of fatty acids, ↓ apoptosis, ↓ oxidative stress, ↓ myocardial inflammation, ↓ fibrosis, ↓ inflammation	Cohen et al.^404^ Kanda et al.^405^ Waldman et al.^406^
		DIO mouse	↑ VEGF, ↑ M2 macrophage	Kim et al.^237^
	CR and IF	Mouse	↓ hyperglycaemia and diacylglycerol in liver	Baumeier et al.^407^
	APF/FMD	Middle-aged mouse Old mouse Human	↓ visceral fat, ↓ IGF-1 levels and PKA activity, ↓ Glucose, ↓ body weight, ↓ CRP, ↑ Ketone body, ↑ IGFBP-1, ↑ MSPC	Brandhorst et al.^238^ Pak et al.^239^
	PR	Mouse	↓ hyperglycaemia, ↓ β cell loss	Laeger et al.^98^
	MR	Mouse	↑ insulin sensitivity, ↑ energy expenditure	Castano-Martinez et al.^408^
	BR	DIO mouse	↑ WAT browning, ↓ adiposity	Ma et al.^247^
	FRD	DIO mouse	↑ levels of branched-chain hydroxy acids, ↑ insulin sensitivity, ↓ hyperglycaemia	Daniel et al.^249^
Cardiovascular diseases	CR	ob/ob mouse	↑ leptin in heart	Sloan et al.^256^
		ob/ob mouse db/db mouse	↓ oxidative stress, ↓ fibrosis, ↓ inflammation, ↓ myocyte hypertrophy	An et al.^257^
	PF	DIO mouse	↑ cardiac vascularity and function	Mishra et al.^260^
	IF	Rat	↓ hypertension pathogenesis	Shi et al.^261^
	FRD	Hypertensive mouse	↑ SCFAs, ↑ circadian rhythm, ↓ cardiac hypertrophy, ↓ fibrosis, ↓ blood pressure	Marques et al.^262^
	KD	Mouse	↑ proliferation of cardiac endothelial cells, ↓ heart hypertrophy	Weis et al.^263^
Intestinal malfunction	CR	Fly Worm	↑ intestinal barrier function, ↑ autophagy, ↑ IRE1/XBP1	Akagi et al.^270^ Luis et al.^271^ Gelino et al.^272^
		Mouse	↑ balanced immunoregulation	Shibolet et al.^409^
	KD	Mouse	↓ inflammation, ↓ ILC3s	Kong et al.^275^
	MR	Mouse	↑ ROS response, ↓ inflammation	Liu et al.^410^
	SR	Mouse	↓ inflammation	Kitamoto et al.^276^
Renal diseases	CR and PR	Mouse	↓ injury post-reperfusion	Robertson et al.^411^
	CR and STF	Mouse	↑ renal function, ↓ injury post-reperfusion, ↓ inflammation	Shushimita et al.^412^
	PR	Mouse	↓ renal ammonia excretion	Lee et al.^279^
	KD	DKD mouse	↑ diabetic albuminuria, ↑ glomerulopathy, ↓ podocyte injury, ↓ senescence	Fang et al.^281^
	FRD	Mouse	↓ renal fibrosis	Marques et al.^262^
	EPA supplement	Mouse	↑ autophagy, ↑ renal ischaemia reperfusion	Yamamot et al.^282^
Nervous system diseases	CR	Mouse Monkey	↑ neurological function	Gräff et al.^284^ Bendlin et al.^285^
		Mouse	↑ neurotrophic factors, ↓ stress response	Vermeij et al^289^ Duan et al.^290^
		Obese mouse	↓ neuronal cell death	Shruthi et al.^288^
		PD mouse	↓ inflammation, ↓ oxidative stress, ↑ remodelled gut microbiota, ↑ protect the substantia nigra and dopaminergic neurons	Zhou et al.^294^
		AD mouse	↑ blood–brain barrier, ↑ cognitive function	Bredesen et al.^296^ Tomi et al.^297^
		HD mouse	↓ striatal human HTT expression, ↓ histone acetylation modifications	Moreno et al.^300^
	CR and KD	Old mouse	↑ memory	Newman et al.^286^ Rojic-Becker et al.^287^
	APF/FMD	middle-aged mouse Old mouse Human	↑ hippocampal neurogenesis, ↑ NeuroD1, ↑ cognitive performance	Sebastian et al.^238^
	Spermidine supplement	Fly	↑ memory and locomotion loss, ↓ senescent mitochondria	Liang et al.^291^
	FRD	Mouse	↑ microglial maturation defects, ↑ synaptic impairments	Liu et al.^292^
	TRF	HD mouse	↑ motor function, ↑ circadian rhythms	Wang et al.
	IF	MS mouse MS human	↓ IL-17 producing T cells, ↑ Treg in gut	Francesca et al.^154^
		Rat	↑ short-term and spatial memory	Shin et al.^413^
		Mouse	↓ neuronal hyperexcitability, ↓ hippocampal synaptic plasticity deficits	Liu et al.^414^
		Mouse	↑ brain inflammation, ↑ neuronal injury	Lazic et al.^415^
Muscle and skeletal diseases	CR	Monkey	↑ muscle structure	Mattison et al.^302^
		Mouse	↑ mitochondrial function, ↑ autophagy	Gutiérrez-Casado et al.^304^
		Rat	↓ muscular apoptosis	Marzetti et al.^305^
		Mouse	↓ oxidative stress	Jang et al.^306^
		Mouse	↑ optimize the proteasome-dependent degradation	Hepple et al.^307^
		Sarcopenia mouse Sarcopenia monkey	↑ muscle mass	van Norren et al.^308^ McKiernan et al.^309^
		Mouse	↑ muscle integrity	Ham et al.^303^
		Rat	↑ bone weakening, ↑ osteoporosis, ↑ bone-healing ability	Villareal et al.^313^ Bodnar et al.^314^
	BCAA supplement	Mouse Human	↑ skeletal muscle hypertrophy	Aoyama et al.^312^
	αKG supplement	Old mouse	↑ osteogenesis, ↑ bone regeneration	Wang et al.^121^
	APF/FMD	Middle-aged mouse Old mouse Human	↓ bone mineral density	Sebastian et al.^238^
Endocrine system diseases	CR	Rat	↓ growth-associated hormones	Trivedi et al.^317^
		Rat	↑ glucocorticoid	Qiu et al.^320^
Respiratory system diseases	CR	Mouse	↑ prevents pulmonary MTB infection	Palma et al.^175^
	KD	Mouse	↑ γδ T cells expansion, ↑ lung barrier functions to resist influenza virus infection	Goldberg et al.^327^
	Tryptophan supplement	Mouse	↑ sensitivity to anti-MTB therapy	Puyskens et al.^324^
	FRD	Mouse	↑ fibre-fermenting bacteria, ↑ SCFAs, ↓ allergic inflammation	Trompette et al.^328^
		Mouse	↑ survival of influenza-infected mice, ↑ functionality of CD8^+^ effector T cells	Trompette et al.^329^
Cancer	CR	Mouse	no benefit in delaying growth or progression of neuroendocrine tumours	Sharp et al.^416^
		Mouse	↓ tumours	Shimokawa et al.^31^
		DIO mouse	↓ proinflammatory cytokines, ↓ angiogenic factors, ↓ tumour metastasis	Sundaram et al.^417^
		Mouse	↑ survival, ↓ metastasis, ↓ IGF-1R, ↓ inflammatory cytokines	Simone et al.^418^
		Mouse	↑ p53, ↓ tumorous growth	Ma et al.^332^
	SRF	Mouse	↓ IGF-1, ↑ ratio of CD8^+^ T/Treg, ↑ efficacy of immunotherapy	Ajona et al.^331^
		Mouse	↓ IGF-1, ↓ Treg, ↑ efficacy of chemotherapy	Pietrocola et al.^90^
		Mouse	↑ efficacy of sorafenib	Krstic et al.^352^
	TRF	DIO mouse	↓ tumour initiation, ↓ obesity-promoted malignant growth, ↓ pulmonary metastasis focuses	Das et al.^330^
	αKG supplement	Mouse	↑ p53, ↓ tumorous growth	Morris et al.^116^
	KD	Mouse	↑ acetoacetate, ↑ growth in mice inoculated with BRAFV600E-mutant melanoma cells	Xia et al.^336^
		Mouse	↑ balanceable proportion of saturated/unsaturated fatty acids, ↓ tumorous stearoyl-CoA desaturase activity, ↓ malignant growth	Lien et al.^339^
		Mouse	↑ efficacy of chemotherapy	Yang et al.^350^
		Mouse	↑ efficacy of immune checkpoint inhibitors	Ferrere et al.^354^
		Mouse Human	↓ ISCs function, ↓ tumorigenesis	Dmitrieva-Posocco et al.^353^
	PUFA supplement	Mouse	↑ ferroptosis, ↓ tumour growth	Dierge et al.^338^
	PR	Mouse	↓ tumour growth	Fontana et al.^419^
		Mouse	↑ response to immunotherapies, ↑ proinflammatory phenotypes, ↓ tumour growth	Orillion et al.^420^
		Mouse	↑APCs and CD8^+^ T cells, ↓ tumour growth	Rubio-Patino et al.^345^
	SR	Mouse	↓ proliferation of T cells, ↓ tumour growth	Ma et al.^421^ Maddocks et al.^340,422^ Sullivan et al.^423^
	MR	Mouse Human	↓ metastasis, ↑ efficacy of chemotherapy ↑ disease-free survival	Gao et al.^216^ Golbourn et al.^424^
	Cystine restriction	Mouse Human	↓ tumour growth, ↑ efficacy of chemotherapy	Wu et al.^351^
	AR	Mouse	↓ ASS1-deficient tumour growth	Poillet-Perez et al.^425^
	Arginine supplementation	Mouse	↓ tumorigenesis ↑ generation of central memory T cells, ↑ survival of T cells	Geiger et al.^426^
	BR	Mouse Human	↓ tumorigenesis, ↓ tumour growth	Li et al.^427^ Thandapani et al.^428^
	Se supplement	Mouse	↑ anticancer properties	Wawrzyniak et al.^69^
	Zn supplement	Mouse	↑ DNA repair function	
	Mg supplement	Mouse	↑ coenzyme function for DNA polymerases	
	FMD	Mouse	↑ efficacy of endocrinotherapy	Caffa et al.^159^
	FRD	Human Mouse	↑ progression-free survival, ↑ efficacy of anti-PD-1-based therapy	Spencer et al.^357^
		Mouse	↑ efficacy of CAR-T cells	Luu et al.^359^
		Mouse	↓ efficacy of CTLA-4 blockade	Coutzac et al.^360^
	NR supplement	Mouse	↑ cell-killing function of CTLs	Wang et al.^361^
		Mouse	↑ efficacy of PD-L1 blockade	Lv et al.^362^
	K^+^ supplement	Mouse	↑ efficacy of CAR-T cells	Vodnala al.^363^

*αKG* α ketoglutarate, *AD* Alzheimer’s disease, *ADF* alternate-day fasting, A*LL* acute lymphocytic leukaemia, *ALP* alkaline phosphatase, *APCs* antigen-presenting cells, *APF* alternating prolonged fasting, *AR* arginine restriction, *ASS1* argininosuccinate synthase 1, *BCAA* branched-chain amino acid, *BR* branched-chain amino acid restriction, *CR* caloric restriction, *CRP* C-reactive protein, *CTL* cytotoxic T lymphocyte, *DIO* diet-induced obesity, *DKD* diabetic kidney disease, *EPA* eicosapentaenoic acid, *EV-D68* Enterovirus D68, *FCR* fermentable carbohydrate restriction, *FMD* fasting-mimicking diet, *FRD* fibre-rich diet, *GGT* gamma-glutamyl transferase, *HD* Huntington’s disease, *IF* intermittent fasting, *IGF-1* insulin-like growth factor 1, *IGF-1R* insulin-like growth factor-1 receptor, *IGFBP-1* insulin-like growth factor binding protein-1, *IL-17* interleukin-17, *ILC3s* innate lymphoid cells group 3, *IRE1* inositol-requiring enzyme 1, *ISCs* intestinal stem cells, *KD* ketogenic diet, *MCT* medium-chain triglycerides, *MD* Mediterranean diet, *MR* methionine restriction, *MS* multiple sclerosis, *MSPC* mesenchymal stem/progenitor cells, *MTB* pulmonary mycobacterium tuberculosis, *NR* nicotinamide riboside, *PD* Parkinson's disease, *PF* periodic fasting, *PKA* protein kinase A, *PR* protein restriction, *PUFA* polyunsaturated fatty acid, *SCFAs* short-chain fatty acid, *SR* serine restriction, *STF* short-term fasting, *Treg* regulatory T cell, *TRF* time-restricted feeding, *VEGF* vascular endothelial growth factor, *WAT* white adipose tissue, *XBP1* X-box binding protein 1.

**Table 4:** The clinical trials of dietary interventions in disease

Disease	Regimen	Effect	Registration number	Reference
Metabolic syndrome	CR	↓ body weight, ↓ reactive oxygen species	NCT00427193 NCT02695511	Redman et al.^255^
		↓ ALP, ↓ GGT, ↑ bilirubin	NCT00427193	Dorling et al.^236^
		↓ body weight, ↓ body fat, ↓ blood pressure	NCT03745612	Liu et al.^244^
	IF	↓ body weight, ↑ insulin sensitivity, ↑ Akkermansiaceae, ↑ Christensenellaceae, ↑ Tanerellaceae	NCT02449148	Sowah et al.^11^
	TRF	↓ oxidative stress, ↓ insulin resistance, ↓ body weight	NCT03867773	Cienfuegos et al.^240^
		↑ cardiometabolic health	NCT03182985	Wilkinson et al.^241^
	Plant-rich diet	alter plasma metabolites, ↓ diabetes risk	Nurses’ Health Study, Nurses’ Health Study II and Health Professionals Follow-up Study	Wang et al.^245^
	MD	↓ cholesterol, ↑ insulin sensitivity, ↓ inflammation, ↑ Faecalibacterium prausnitzii	NCT03071718	Meslier et al.^246^
	FRD	↑ insulin sensitivity	NCT03477916	Mocanu et al.^248^
	FRD and excise	↓ body weight, ↓ cholesterol level in plasma and liver, ↑ glucose tolerance	NCT03852069	Rodriguez et al.^250^
Cardiovascular diseases	CR	↓ heart rate and blood pressure	NCT00427193 NCT02695511	Redman et al.^255^
	PF	↓ blood pressure	NCT02099968	Maifeld et al.^196^
	MD	microbial changes, ↓ body weight, ↓ cardiometabolic biomarkers	NCT03020186	Rinott et al.^264^
		↓ atherosclerosis, ↓ cardiovascular events	NCT00924937	Delgado-Lista et al.^266^ Jimenez-Torres et al.^267^
		alter functional and components of the gut microbiome	Health Professionals Follow-up Study	Wang et al.^265^
Intestinal malfunction	FRD	↓ inflammation	NCT04147598	Fritsch et al.^274^
	FCR	↑ functional gastrointestinal symptoms	U.K. tertiary IBD center	Prince et al.^273^
Renal diseases	AGEs restriction	↑ balance gut microbiota	NCT02467530	Yacoub et al.^280^
	MD	↑ kidney function	NCT00924937	Podadera-Herreros et al.^283^
Nervous system diseases	MCT supplement	↑ cognitive and gait functions	UMIN000033447	Mutoh al.^298^
	NR	↓ inflammation	NCT03816020	Brakedal et al.^295^
	MD	↑ cognitive function, ↓ inflammation, microbiome alterations	NCT01754012	Ghosh et al.^299^
	IF	↓ IL-17 producing T cells, ↑ Treg in gut	NCT02411838	Cignarella et al.^154^
Endocrine system diseases	CR	↓ energy expenditure, ↓ thyroid axis activity	NCT00427193 NCT02695511	Redman et al.^255^
		↓ mammary-gland and ovulation	IRCT20140907019082N9	Tabrizi et al.^321^
		↑ testosterone	–	Schulte et al.^322^
	ADF	↓ circulating fT3, ↑ parathyroid hormone	NCT02673515	Stekovic et al.^80^
Respiratory system diseases	CR	↑ improve dyspnoea and obstruction symptoms	ACTRN126000056897	McDonald et al.^325^
	KD	↑ respiratory function	–	Rubini et al.^326^
Cancer	KD-IF	↓ leptin and insulin, ↑ uric acid	NCT01754350	Voss et al.^337^
	Asparaginase supplement	agent in chemotherapy of ALL	NCT03987542	Gottschalk et al.^429^
	FMD	↓ Treg and immunosuppressive myeloid, ↑ CD8^+^ T cell, ↑ response to immunotherapies	NCT03340935	Vernieri et al.^344^
		↑ efficacy of neoadjuvant chemotherapy	NCT02126449	de Groot et al.^348^
	FRD	↓ risk of lung cancer	–	Yang et al.^356^
	Prebiotics supplementation	↑ efficacy of immune checkpoint inhibitors	NCT03829111	Dizman et al.^358^

*ADF* alternate-day fasting, *AGEs* advanced glycation end products, *ALL* acute lymphocytic leukaemia, *ALP* alkaline phosphatase, *CR* caloric restriction, *FCR* fermentable carbohydrate restriction, *FMD* fasting-mimicking diet, *FRD* fibre-rich diet, *GGT* gamma-glutamyl transferase, *IF* intermittent fasting, *IL-17* interleukin-17, *KD* ketogenic diet, M*CT* medium-chain triglycerides, *MD* Mediterranean diet, *NR* nicotinamide riboside, *PF* periodic fasting, *Treg* regulatory T cell, *TRF* time-restricted feeding.

### Metabolic syndrome

Overnutrition contributes to accelerate disease progression, notably with obesity and type 2 diabetes. A myriad of diseases, including non-alcoholic fatty liver disease (NAFLD), diabetes, cardiovascular disease (CVD), and cancer, are positively correlated with obesity.^230^ A fat-rich diet and excessive intake of BCAAs, tryptophan and its metabolites, and methionine result in weight gain and obesity-associated disease (reviewed in ref. ^231^) For example, a mixed-protein diet mimicking a Western diet exacerbates insulin resistance and obesity. Mechanistically, the mixed protein source can give rise to elevation of acylcarnitines and microbially generated BCAAs in plasma and liver, further activating mTORC1/S6 kinase 1 (S6K1) to increase glucose synthesis in hepatocytes.^232^ As a novel diet-dependent obesogen, microbial δ-valerobetaine (VB) is found to exacerbate visceral fat mass and phenotypic obesity by inhibiting mitochondrial fatty acid oxidation.^233^

Obesity comprehensively leads to comorbidities. Thus, dietary intervention is a promising method to combat these disorders. Specifically, CR has the capability to polarize M2 macrophages and recruit eosinophils in fat to promote beige fat within the visceral and subcutaneous fat tissue, consequently contributing to metabolic improvements and lipopenia in mice.^234^ Likewise, CR induced a high release of adiponectin to impact adiposity by activating PGC-1α, SIRT1, and NAMPT in adipose tissues.^235^ In clinical, CR is observed to reduce the levels of alkaline phosphatase (ALP) and gamma-glutamyl transferase (GGT) in health adults to improve liver function.^236^ Moreover, diet patterns are pivotal in obesity-related metabolic dysfunction. In WAT, isocaloric IF is capable of polarizing macrophage alternative activation via induction of vascular endothelial growth factor (VEGF) expression, then improving metabolic dysfunction primarily through adipose thermogenesis.^237^ Alternating prolonged fasting (PF) has been found to reduce high-risk factors for cancer and ageing-associated diseases in the absence of obvious detriments in a pilot clinical trial.^238,239^ Compared to controls, TRF leads to a considerable decrease in oxidative stress, insulin resistance and body weight^240^ and improves cardiometabolic health for patients with metabolic syndrome.^241^ Similarly, an every-other-day fasting (EODF) regimen prominently mitigates insulin resistance, NAFLD and obesity. EODF not only increases mitochondrial protein content and fatty acid synthesis enzymes in WAT^242^ but also accelerates the intake of microbially fermented acetate and lactate in beige cells by electively upregulating their transporters.^243^ However, the benefits of TRF on loss of body fat and body weight are not superior to that of daily CR in obese patients.^244^ Ultimately, alteration of nutritive components is a feasible way to mitigate metabolic syndrome. The Mediterranean diet (MD) and plant-rich diets are associated with a lower risk of type 2 diabetes in healthy adults.^245,246^ BCAA restriction has the capacity to improve health in obese and non-obese animals as well as in humans.^165^ Likewise, branched-chain keto acids (BCKAs) have been found to suppress inguinal WAT browning and thermogenesis to expedite high-fat diet-induced obesity, and telmisartan significantly reduced BCKA levels to result in enhanced WAT browning and reduced adiposity.^247^ In addition, daily high-fibre supplementation combined with faecal microbiota transplantation (FMT) or yogurt consumption alleviates insulin resistance in cases with metabolic syndrome or severe obesity.^248,249^ In similar, daily high-inulin supplementation combined with excise are able to reduce cholesterol level in plasma and liver, improve glucose tolerance and lead to weight loss through augmentation of inulin-degrading bacteria in gut.^250^ In contrast, a CNOT6L inhibitor elevates hepatokine FGF21 and growth differentiation factor 15 (GDF15) levels by stabilizing their mRNAs and resulted in a remarkable improvement of overnutrition-induced metabolic syndrome.^251^

Although there is clearly a relationship between nutritional intervention and metabolic disorders, the type and pattern of diet may be vital. A case-by-case analysis is required to demonstrate how nutrition interventions are regarded as adjuvant treatments for metabolic syndromes.

### Cardiovascular diseases

Diet is closely linked to the onset of cardiovascular health and disease, and cardiovascular health has frequently focalized dietary interventions.^252^ High-fat/salt diets and diets extremely high in BCAAs are high-risk factors for CVD.^253^ For example, dietary BCAA intake significantly facilitates platelet activation, further increasing the risks of arterial thrombosis and cardiovascular disease.^254^ Recently, CR is found to reduce adverse factors for CVD and improve the parameters of cardiac function, such as energy expenditure, heart rate and blood pressure.^255^ One possible mechanism is that CR reduces free leptin in plasma, which reverses myocardial hypertrophy and reduces lipid accumulation.^256^ CR also reduces oxidative stress, fibrosis, cardiac inflammation and myocyte hypertrophy in mice.^257^ Studies have further demonstrated the advantages of CR, such as lessened triglyceride, cholesterol and LDL levels.^258,259^ A 5-day fast reduces blood pressure by altering the gut microbiome and systemic immune status.^196^ In obese models induced by a fat-rich diet, a 5-day fasting-mimicking diet (FMD) is able to promote visceral and subcutaneous fat loss and protect against obesity-impaired cardiac vascularity and function.^260^ Similar to CR, IF retards hypertension pathogenesis in rats through alteration of the metabatic profile in the caecum and plasma and the composition of the gut microbiota.^261^ Considering food composition, high fibre consumption is discovered to elevate the richness of SCFA-generated microbial communities to include Bacteroides acidifaciens. SCFA supplementation as well as a high-fibre diet stabilize circadian rhythm and downregulate Egr1, a master cardiovascular regulator, in the heart and kidney and are conducive to decreasing cardiac hypertrophy and fibrosis as well as blood pressure.^262^ A low-carbohydrate, high-fat KD elevated the levels of ketone bodies to stimulate the proliferation of cardiac endothelial cells and prevent heart hypertrophy.^263^ Particularly, MD alters the microbial compositions and function to benefit cardiometabolic homoeostasis,^264,265^ and reduce diverse cardiovascular events like atherosclerosis to prevent cardiovascular disease.^266,267^ Altogether, dietary interventions partially mediate these cardiovascular complications.

### Intestinal malfunction

Diet has been discovered to profoundly affect intestinal functions. Digestive disorders such as colitis and Crohn disease are regulated by nutritional therapies. Some diet plans are adverse for intestinal function. For instance, long-term red meat consumption has been related to a high risk of digestive disease. The N-glycolylneuraminic acid (Neu5Gc) enriched in red meat can only be synthesized by gut microbiota. The red-meat diet induces an increase in *Bacteroidales* and *Clostridiales* to produce Neu5Gc, which is a potential factor contributing to the inflammation-mediated promotion of diseases and carcinomas.^268^ In active human Crohn disease, ω−3 and ω−6 PUFAs induce chemokine expression in epithelial cells by activating toll-like receptor 2 (TLR2)/inositol-requiring enzyme 1α (IRE1α) in epithelial cells.^269^ Some nutrition strategies are enabled to sustain intestinal homoeostasis. First, dietary restriction is demonstrated to improve intestinal barrier function and maintain intestinal homoeostasis via upregulation of Myc^270^ and activation of IRE1/X-box binding protein 1 (XBP1) endoplasmic reticulum (ER) stress signalling,^271^ excitation of autophagy in worms^272^ and alteration of the intestinal microbiome.^11^ Specific dietetic techniques for dietary restriction of fermentable carbohydrates and CR have been discovered to improve functional gastrointestinal symptoms and combat the worsening of digestive disorders.^273^ In contrast, the effects of a high-fibre, low-fat diet reduces inflammatory markers and reverses intestinal dysbiosis to benefit patients with ulcerative colitis.^274^ In addition, KD has been found to be able to protect the function of the intestinal barrier and alleviate colitis. Mechanistically, KD contributes to a reproducible increase in *Akkermansia* but declines in *Escherichia*/*Shigella*, subsequently eliminating inflammatory factors (Ccl4, IL-17α, IL-18, IL-22) and restraining the differentiation of group 3 innate lymphoid cells (ILC3s) and related inflammatory cytokines (IL-17α, IL-18, IL-22, Ccl4).^275^ Moreover, L-serine restriction markedly reduces the abundance and availability of *E. coli* LF82, which catabolizes L-serine and blunts the inflammatory response.^276^ Overall, suitable dietary patterns appear beneficial to properly sustain metabolic and barrier functionality in the gut, but the detailed mechanisms should be further deciphered.

### Renal diseases

Renal functionality is broadly dependent on the state of nourishment. Notoriously, overnutrition impairs renal function. For example, dietary protein aggravates progression to chronic kidney disease (CKD) in mice. A high-protein diet elevates levels of uraemic toxins such as microbial indoxyl sulfate, indole and hydrogen sulfide (H2S).^277^ Likewise, excessive salt intake has been suspected to exacerbate the symptoms of renal diseases via multiple mechanisms (reviewed in ref. ^278^) Consequently, appropriate alimentative methods alleviate renal dysfunction. Recent studies in murine models have indicated that dietary protein restriction decreases renal ammonia excretion by altering ammonia metabolism.^279^ In peritoneal dialysis, the restriction of dietary advanced glycation end products (AGEs) remodels gut-microbiome composition, suggesting that a reduction in AGEs potentially alleviates CKD progression.^280^ Streptozotocin is most often used to elicit diabetic kidney disease (DKD) in murine models. In these mouse models, the replenishment of β-hydroxybutyrate inhibits glycogen synthase kinase 3β (GSK3β) but activates Nrf2 to improve diabetic albuminuria and glomerulopathy and substantially mitigate podocyte injury and senescence.^281^ Similarly, acetate generated in a high-fibre diet markedly reduced renal fibrosis.^262^ Some studies have uncovered the possible mechanisms by which nourishment may impact renal ischaemia-reperfusion, showing that nutritive intervention has therapeutic potential.^282^ In clinical, coronary heart disease patients with T2DM undergo a MD for 5 years, and they exhibit a remarkably decline of creatinine-based glomerular filtration rate, suggesting that MD could preserve kidney function.^283^ Taken together, these studies confirm that optimized diets serve as a dominant factor for relieving renal dysfunction.

### Nervous system and neurodegenerative diseases

Due to potential neuroprotection, optimal diet has received serious attention. First, declines in neurological function are able to be delayed by dietary restriction in mice^284^ and macaques,^285^ which may be attributed to SIRT1 activation. CR and KD improve late-life memory in mice.^286,287^ In obese rats, CR is capable of accelerating proteasome-dependent degradation and ameliorating neuronal cell death to protect overall neuronal function.^288^ Furthermore, CR stimulates the secretion of neurotrophic factors and relieves diverse stress responses as well as DNA maintenance in mice.^289,290^ Added to the diet cycle and components, FMD contributes to improved cognitive performance in aged mice and in humans through elevation of NeuroD1 but reduced IGF-1 levels and PKA activity in hippocampal neurogenesis.^238^ In the Drosophila brain, dietary spermidine induces the levels of eIF5A hypusination, which reverses age-associated memory and locomotion loss and rescues the homoeostasis of senescent mitochondria.^291^ Fortuitously, the behaviour and gut microbiomes of offspring can be disrupted by maternal obesity. SCFA supplementation or a high-fibre diet in either offspring or mothers alleviates microglial maturation defects and synaptic impairments.^292^ In summary, favourable diet strategies have the capacity to align the function of the nervous system for health improvement.

Consequently, an increasing number of studies have focused on the beneficial effects of diet in treating neurological diseases.^293^ Initially, CR suppresses inflammation and oxidative stress, remodels gut microbiota, and protects the substantia nigra and dopaminergic neurons, which is conducive to relieving symptoms of Parkinson disease (PD).^294^ In addition, oral NR to replenish NAD^+^ elevates the level of cerebral NAD^+^, which reduces inflammatory factor levels in both cerebrospinal fluid and plasma. Thus, NR supplementation is a safe and effective treatment for PD.^295^ Nutritional intervention also benefits the symptoms of AD. The evidence shows that dietary intervention during neurodegenerative treatments enables relief of the AD process.^296^ CR in early-life rats preserves the blood–brain barrier to delay AD pathologies.^297^ Comparably, MD as well as the supplementation with medium-chain triglycerides (MCT) improves gait and cognitive functions in healthy older adults.^298,299^ As a neurodegenerative disease, Huntington disease (HD) is distinguished by protein aggregates in the brain, and CR advantageously functions in HD mice via histone acetylation modifications.^300^ Similarly, TRF enables perfect motor function and circadian rhythms in HD mice.^301^ Finally, multiple sclerosis (MS) is a common CNS autoimmunity. IF results in increased richness of gut bacterial, enhancement of antioxidative microbial metabolic pathways, and alteration of T-cell subtype proportions in the gut to ameliorate the clinical course and pathology of MS.^154^ It is evident that alimentative strategies profoundly affect neurological health and disease, and future studies should utilize diet as a promising therapy for neurological disease.

### Muscle and skeletal tissue

Muscle is regarded as one of the main metabolic organs, and muscle homoeostasis is dramatically mediated by food intake. It is evident that CR preserves muscle structure in late life^302^ and improve the muscle integrity in aged mouse.^303^ From a mechanistic perspective, CR can improve mitochondrial function and autophagy to provide muscular benefits.^304^ CR prevents muscular apoptosis by upregulating IL-15.^305^ Additional targets for CR are found to reduce oxidative stress for persistent advantages in muscle tissue.^306^ CR also optimizes proteasome-dependent degradation to sustain muscle health in mice.^307^ Considering CR-induced superiority, diet intervention is frequently applied to determine its efficacy in muscle-associated disease. In the early stages of senescence-related sarcopenia, CR sustains muscle mass in mice^308^ as well as in monkeys.^309^ Regrettably, CR has no effect on muscle health in patients with sarcopenia.^310^ What is more, the influences of fasting on muscle repair are uncovered. During fasting or KD, muscle stem cells (MuSCs) are converted to highly resilient deep quiescence where they could obtain ameliorative survival under diverse cellular stress.^311^ However, patients ingesting dietary proteins mainly at breakfast were found to have higher muscle function than those ingesting dietary proteins at dinner. In the early active phase, protein intake, especially BCAA supplementation, promotes skeletal muscle hypertrophy in a muscle-clock-reliable manner.^312^ Although the results thus far have been unpromising in clinical trials, a multitude of investigations into how the rhythm and pattern of diets influence human health are needed.

Bone health highly depends on a balanced diet, particularly with regard to dietary mineral intake. Unexpectedly, CR has been demonstrated to increase the risk of bone weakening and osteoporosis^313^ and reduce bone-healing ability in catagmatic or osteoporotic rats.^314^ A potential reason may be a decrease in mineral intake. Conversely, CR paired with exercise or mild CR is able to improve bone health.^315,316^ The administration of αKG ameliorates osteoporosis in aged mice. In detail, αKG promotes the proliferation, colony formation, migration, and osteogenesis of bone marrow mesenchymal stem cells (MSCs) by demethylating H3K9me3 and H3K27me3 to increase Nanog expression and BMP signalling. Activated MSCs attenuate age-related bone loss by promoting osteogenesis and accelerating bone regeneration.^121^ In general, meticulous dietary adjustments should be closely explored to strengthen bone health.

### Endocrine system

The endocrine system regulates the effectors of nutritional intervention, but dietary intake influences hormonal signalling. Therefore, the interactions between endocrine modulation and diets are quite complicated. Endocrine-associated metabolic pathways and CR are intricately connected and mainly involve ghrelin, leptin, adiponectin, FGF21, GDF15, ACBP and asprosin. For example, ghrelin, as a hunger hormone, is acylated to stimulate glycogenolysis in response to CR.^317^ Following long-term CR, growth-associated hormonic pathways are observably repressed in rats.^318^ The hypothalamic/pituitary axis is heavily influenced by nutrients.^319^ Diet-mediated alterations in hormones and cytokines occur in multiple neuroendocrine axes. In the adrenal glands, CR amplifies the neuroprotective effects of glucocorticoids.^320^ CR has been found to inhibit hormone secretion in mammary glands and ovulation in females to perturb signal transduction between the pituitary gland and gonads.^321^ Likewise, testosterone levels are elevated in men by CR.^322^ Fasting induces profound changes in the hypothalamic-pituitary-thyroid (HPT) axis. In a clinical trial, long-term ADF leads to reduced secretion of circulating fT3 and high release of parathyroid hormone (PTH) but sustains normal function of the thyroid gland.^80^ Similarly, fasting leads to phosphorylation of thyroid hormone receptor β 2 isoform (THRB2) at the S101 site via AMPK/cyclin-dependent kinase 2 (CDK2) activation, and repress the functions of thyroid-stimulating hormone and thyrotropin-releasing hormone through negative feedback.^323^ Overall, the disparity and the interplay in the endocrine system exhibit a systemic function of diet and demonstrate that nutritional strategies extensively affect endocrine regulation and that these hormones are pivotal for determining applicable diet-based therapies for endocrine disorders.

### Respiratory system

Diet profoundly impacts health and disease in the respiratory system. Originally, CR is found to prevent pulmonary mycobacterium tuberculosis (MTB) infection. Mechanistically, CR impairs both fatty acid β-oxidation and mTOR activity but induces glycolysis and autophagy in immune cells. As a result, CR reduces the bacterial load and consolidated the MTB reservoir in foam cells to restrain lung damage and endow lung epithelial cells with an intact barrier capable of confining spreading.^175^ After infection of Enterovirus D68 (EV-D68), a respiratory virus, fasting is observed to attenuate virus replication through stimulating of autophagic flux.^324^ Furthermore, dietary tryptophan affects sensitivity to anti-MTB therapy. Tryptophan catabolites bind and activate aryl hydrocarbon receptor (AhR), which combines with rifampicin (RIF) and rifabutin (RFB), anti-MTB drugs. Subsequently, they function to increase drug metabolism and reinforce the host antibacterial response to consolidate the efficacy of anti-MTB drugs.^324^ In obese patients with chronic obstructive pulmonary disease (COPD), CR paired with exercise is capable of improving dyspnoea and obstruction symptoms.^325^ Regarding food composition, a low-carbohydrate, high-fat KD is discovered to potentially preserve respiratory function through a decrease in the storage of carbon dioxide in the lungs.^326^ Meanwhile, KD remodels the metabolic process of γδ T cells and contributes to γδ T-cells expansion, which improves lung barrier functions to resist influenza virus infection.^327^ In addition, dietary fibre has a vital effect on lung functions. A fibre-rich diet augments the proportion of fibre-fermenting bacteria in both the lung and gut to elevate circulating levels of SCFAs, subsequently leading to a decline in allergic inflammation in the lung. SCFAs reinforce the production of dendritic cells (DCs) and macrophage precursors in bone marrow haematopoiesis, and DCs with high phagocytic capacity migrate to the lungs to promote the effector function of Th2 cells. Likewise, SCFAs bind to G protein-coupled receptor 41 (GPR41) to alleviate allergic inflammation.^328^ In addition, a high-fibre diet prolongs the survival of influenza-infected mice. The main mechanism is that SCFAs facilitate the functionality of CD8^+^ effector T cells, and also induce the polarity of macrophages by enhancing precursor generation in bone marrow haematopoiesis. These macrophages restrain the secretion of the chemokine CXCL1, which recruits neutrophils in the airways.^329^ Initial studies highlights that the manner of nutritional therapy is key to improving the respiratory microecosystem and function for lung health.

### Cancer

Dietotheroapy is a prime adjunctive therapy for cancer treatment involving multiple intrinsic and extrinsic pathways. In intrinsic mechanisms, nutrient interventions are capable of suppressing neoplastic growth and metastasis. In a mouse model, TRF is found to delay tumour initiation, retard obesity-promoted malignant growth and decrease pulmonary metastatic foci, partially dependent on insulin levels and metabolic state.^330^ In addition, CR and FMD have been found to reduce IGF-1 levels to delay cancer risk.^90,138,331^ CR is capable of upregulating p53, a tumour suppressor, in mice.^332^ Similarly, GR deacetylates and degrades mutant p53 in an autophagy-dependent way. CR highly stimulates SIRT1 in mice with p53 deficiency, suggesting that deacetylation of mutant p53 results in a reduced level.^333^ αKG has the capacity to repress tumour growth depending on p53 status, while succinate has been found to competitively inhibit the effect of αKG to abrogate p53-mediated tumour suppression. The accumulation of αKG is able to increase 5-hydroxymethylcytosine (5hmC) modification in chromatin, accompanied by tumour cell differentiation and decreasing tumour cell fitness.^116^ mTOR and FOXO3 are additional mechanisms found to be associated with CR and tumour suppression.^93^ Ketone bodies derived from stromal cells can be absorbed by tumour cells to exhibit metabolic adaptation and an aggressive phenotype.^334^ Similarly, β-hydroxybutyrate is an alternative cell-intrinsic or systemic fuel that can promote the progression of pancreatic ductal adenocarcinoma.^335^ A fat-enriched KD elevates the circulatory levels of acetoacetate, which expedites malignant growth in mice inoculated with BRAF^V600E^-mutant melanoma cells;^336^ whereas KD combined with IF leads to an increase of uric acid but reduction of leptin, insulin and glucose in patients with recurrent brain tumours.^337^ Conversely, ω−3 and ω−6 PUFAs selectively induces ferroptosis in malignant cells, and an ω−3 PUFA-enriched diet dramatically decelerates murine tumour growth.^338^ Consistently, CR as well as KD with altering fat composition slows malignant growth via an imbalanced ratio of saturated to unsaturated fatty acids cooperating with decreasing tumorous stearoyl-CoA desaturase activity.^339^ Thus, the specific species of fatty acid determine whether KD impairs tumour growth. Dietary restriction of protein and special amino acids, such as serine, methionine and BCAAs, can retard tumour growth (reviewed in ref. ^93^) For example, an improved survival during concomitant dietary restriction of serine and glycine is found in mouse models of multiple tumour types.^340^ Moreover, a series of drugs mimicking amino acid restriction have been put forward. Systemic cysteinase enables to deplete cysteine and inhibit tumour growth through elevating ROS and inducing tumour-selective ferroptosis.^341,342^ Glutamine antagonism in tumour-bearing mice exhibits forceful antitumor effects by down-regulating oxidative and glycolytic metabolism of malignant cells but dramatically promoting oxidative metabolism of effector T cells.^343^ In mineral metabolism, Se replenishment has antioxidant and DNA repair effects to exert anticancer properties. As a cofactor in DNA repair genes, zinc is shown to perform a DNA repair function through estimation of oxidized guanine. Similarly, magnesium exerts coenzyme functions for diverse DNA polymerases.^69^ In the extrinsic pathways, short-term fasting induces the depletion of regulatory T cells (Tregs) in mice, thereby improving anticancer immunosurveillance.^90,331^ In a clinical trial, FMD contributes to better clinical outcomes in patients with cancer. Mechanistically, FMD decreases the ratio of Tregs to immunosuppressive myeloid cells in peripheral areas and strengthens intratumoral cytotoxic effectors to profoundly remodel antitumor immunity.^344^ Considering the nutrient proportions, a low-protein diet reduces tumour growth through expansion of antigen-presenting cells (APCs) and CD8^+^ T cells.^345^ Unexpectedly, abnormal accumulation of potassium ions (K^+^) in the tumour interstitial fluid has been found to contribute to impaired T-cell effector function through suppression of AKT-mTOR signalling.^346^

Dietary regulation also influences responsiveness to cancer treatments (which has been reviewed in ref. ^347^) A short-term fasting or FMD synergizes with chemotherapy to inhibit tumour growth by enhancing CD8^+^-dependent tumour cytotoxicity or chemotherapy-induced DNA damage.^90,348,349^ KD potentiates cytotoxic chemotherapy in the treatment of tumours in murine pancreatic cancer;^350^ by contrast, dietetic cystine restriction retard tumour growth and enhance the efficiency of chemotherapy in colon cancer model.^351^ In a murine hormone-receptor-positive breast cancer model, PF or FMD reinforces the efficiency of endocrine therapeutic drugs such as fulvestrant and tamoxifen in mice and humans. Furthermore, a FMD combining fulvestrant and CDK4/6 inhibitor causes extensive tumour regression and reversed drug resistance.^159^ In targeted therapy, fasting synergizes with the efficacy of sorafenib in hepatocellular carcinoma accompanied by acquired resistance to sorafenib.^352^ Immunotherapy is particularly strengthened with diverse eating strategies in mouse models. Short-dated fasting decreases the serum levels of IGF-1 and IGF-1 receptor expression in neoplastic cells and potentiates the therapeutic effect of PD-1 inhibitors to suppress malignant progression.^331^ Similarly, picropodophyllin, as an inhibitor of IGF-1R, stimulates the infiltration of intratumoral cytotoxic T lymphocytes (CTLs) but reduces Tregs to enhance the efficacy of chemoimmunotherapy with a combination of immunogenic chemotherapeutics and PD-1 antagonists.^138^ KD is capable to suppress colorectal tumorigenesis,^353^ and KD or oral 3-hydroxybutyrate supplementation re-establishes therapeutic responses to immune checkpoint inhibitors.^354^ Mechanistically, ketone bodies profoundly impact human T-cell responses by markedly enhancing the capacity of CD4^+^ T cells, CD8^+^ T cells, and Tregs and augmenting the formation of memory T cells.^355^ In addition, dietary consumption enriched in fibre and yogurt is associated with a low risk of lung cancer.^356^ A fibre-rich diet as well as prebiotics supplementation prolongs progression-free survival and synergizes anti-PD-1-based therapy in mice.^357,358^ In syngeneic murine cancer models, SCFA supplementation heightens the anticancer capacity of ROR1-targeting CAR-T cells and antigen-specific CTLs.^359^ However, high SCFA levels limit the accumulation of memory T cells and tumour-specific T cells as well as the expression of ICOS on T cells and CD80/CD86 on DCs to resist CTLA-4 blockade.^360^ For dietary microelements, replenishing NAD^+^ boosts T-cell-based immunotherapy by facilitating the cell-killing function of CTLs.^361^ In multiple types of tumours, the biogenesis of NAD^+^ upregulates PD-L1 expression within IFNγ stimulation and prompts tumour immune escape in a CTL-dependent manner. Therefore, anti-PD-L1 treatment is more effective in tumours with high NAMPT, and NAD^+^ supplementation augments the efficacy of PD-L1 blockade in tumours resistant to immunotherapy.^362^ Another study also indicates that a high concentration of K^+^ in the tumour microenvironment (TME) is able to suppress the tumour-killing function of CTLs but had no effect on T-cell proliferation by restraining CTL nutrient absorption.^363^ However, a high K^+^ concentration improves the anticancer activity, pluripotency and durability of adoptively transferred CD8^+^ T cells by reprogramming their metabolic processes.^363^ Thus, potassium in the TME reshapes T-cell metabolism to enhance anticancer immunological reactions. Taken together, proper adjustment of the manner and composition of diet not only decelerates tumour progression but also potentiates the therapeutic effect of diversified antineoplastic strategies.

## Conclusion

Notably, nutritive intervention is viewed as an efficient therapy for counteracting morbidities and maintaining health. However, there are several unsolved questions about dietary functionality and its clinical application. First, future studies should elaborate the period and circadian rhythm of dietary treatment, such as curative endurance, cycles or meal times. Moreover, future studies should focus on the mechanisms by which strict nutrient constituents such as lipids, carbohydrates, amino acids, micronutrients and metabolites modulate morbid progression and health state. It is crucial to understand how to combine diet with sleep, exercise and other therapeutic interventions to acquire cooperative effects that improve healthspan at the tissue and organismal levels. Considering that gene variants like Pkn involved in nutrition-mediated response,^364^ the genetic disparity should be defined in personalized nutrition intervention. The nutritive functions in diverse genotypes also require further exploration. Due to the heterogeneity within individuals in response to dietary interventions, biomarker panels need to be developed, and diet-based therapeutic strategies need to be optimized. Similarly, it has been reported that diet-mediated functions exhibit sexual disparity in diverse organisms including humans.^365,366^ Therefore, gender necessitates consideration in nutrient-based therapies. Because demographic factors such as age and sex and genotypes are currently not taken into consideration in diet-based trials, successive studies need to elucidate their pivotal roles based on multi-omics data analysis and systems biology.

Considering that nutrient restriction is unattainable in humans, replacement therapies that in part imitate the advantages of dietary intervention at the physiological and molecular levels are prospective. This approach requires seeking behavioural interventions or compounds that serve as mimetics and inducers of alimentation intervention, such as CRMs.^90,137,138,140^ It is hopeful that individualized diets could potentially delay disease progression and maximize health maintenance.
